# Unraveling Electron Dynamics in p-type Indium Phosphide (100): A Time-Resolved Two-Photon Photoemission Study

**DOI:** 10.1021/jacs.3c12487

**Published:** 2024-03-19

**Authors:** Jonathan Diederich, Jennifer Velasquez Rojas, Mohammad Amin Zare Pour, Isaac Azahel Ruiz Alvarado, Agnieszka Paszuk, Rachele Sciotto, Christian Höhn, Klaus Schwarzburg, David Ostheimer, Rainer Eichberger, Wolf Gero Schmidt, Thomas Hannappel, Roel van de Krol, Dennis Friedrich

**Affiliations:** † Institute for Solar Fuels, Helmholtz-Zentrum Berlin für Materialien und Energie GmbH, Berlin 14109, Germany; ‡ Institut für Physik, Technische Universität Ilmenau, Ilmenau 98693, Germany; § Lehrstuhl für Theoretische Materialphysik, Universität Paderborn, Paderborn 33095, Germany; ∥ Institut für Chemie, Technische Universität Berlin, Berlin 10623, Germany

## Abstract

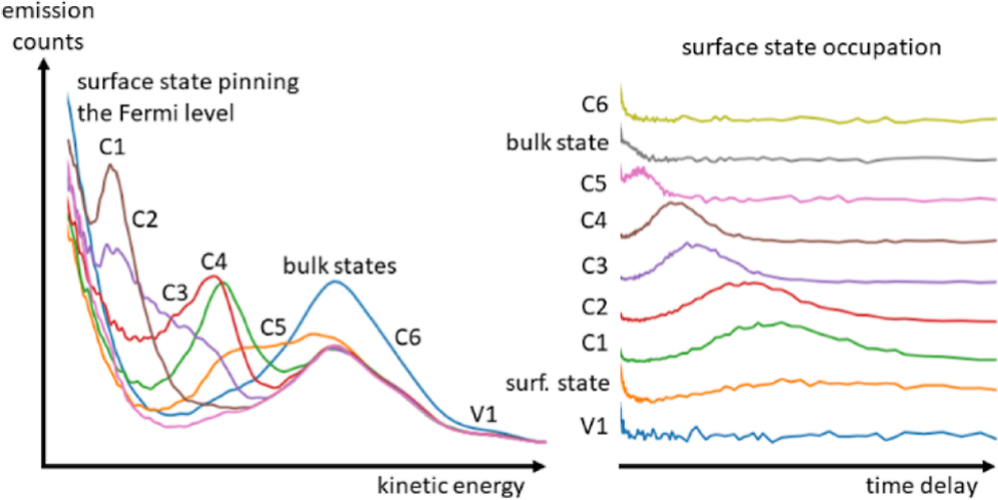

Renewable (“green”) hydrogen production through direct photoelectrochemical (PEC) water splitting is a potential key contributor to the sustainable energy mix of the future. We investigate the potential of indium phosphide (InP) as a reference material among III–V semiconductors for PEC and photovoltaic (PV) applications. The p(2 × 2)/c(4 × 2)-reconstructed phosphorus-terminated p-doped InP(100) (P-rich p-InP) surface is the focus of our investigation. We employ time-resolved two-photon photoemission (tr-2PPE) spectroscopy to study electronic states near the band gap with an emphasis on normally unoccupied conduction band states that are inaccessible through conventional single-photon emission methods. The study shows the complexity of the p-InP electronic band structure and reveals the presence of at least nine distinct states between the valence band edge and vacuum energy, including a valence band state, a surface defect state pinning the Fermi level, six unoccupied surface resonances within the conduction band, as well as a cluster of states about 1.6 eV above the CBM, identified as a bulk-to-surface transition. Furthermore, we determined the decay constants of five of the conduction band states, enabling us to track electron relaxation through the bulk and surface conduction bands. This comprehensive understanding of the electron dynamics in p-InP(100) lays the foundation for further exploration and surface engineering to enhance the properties and applications of p-InP-based III–V-compounds for, *e.g.*, efficient and cost-effective PEC hydrogen production and highly efficient PV cells.

## Introduction

Renewable hydrogen plays a crucial role in achieving a sustainable energy mix for the future. Due to the 50–100 times lower current densities and efficient cooling of the absorbers, green hydrogen production with direct photoelectrochemical (PEC) water splitting offers some compelling advantages over conventional PV-electrolyzer systems.^1^ To date, III–V semiconductors have demonstrated the highest solar-to-hydrogen conversion efficiencies.^2,3^ Their direct band gaps, high electron mobilities, and low exciton binding energies in combination with good surface- and band gap tunability make them highly attractive for PEC and PV applications. In our study, we focus on InP(100) as a reference material among P-containing III–V semiconductors such as GaP, GaInP, or AlInP, which all display analogous surface reconstructions.^4−6^ InP is a well-established semiconductor used for PEC^3,7−12^ with various known surface reconstructions. Under PEC operating conditions using an aqueous electrolyte, the choice of InP surface reconstruction influences its surface electronic energy levels, surface charge carrier lifetimes and stability, and thus its overall performance.^13−16^ For InP(100), depending on the chemical potentials of P and H_2_, typically two surface reconstructions are possible: (i) the phosphorus-terminated “P-rich” p(2 × 2)/c(4 × 2) surface reconstruction consists of buckled P–P dimers stabilized by one H atom each.^17,18^ The H atom is required to satisfy the electron counting principle predicted by Hahn and Schmidt^13,17^ and verified experimentally in several studies.^4,19,20^ The adjacent buckled phosphorus dimer rows can be arranged in-phase, forming a p(2 × 2) surface reconstruction, or out-of-phase, forming a c(4 × 2) surface reconstruction. A mix of domains consisting of each of these reconstructions results in the (2 × 1) low-energy electron diffraction (LEED) pattern observed for this surface; thus, the P-rich surface is commonly described as the “(2 × 1)-like” surface. The (2 × 1)-like p(2 × 2)/c(4 × 2) reconstruction is only observed when prepared under hydrogen exposure such as in metalorganic vapor phase epitaxy (MOVPE) or chemical beam epitaxy. In contrast, rather disordered surfaces are observed in methods such as molecular beam epitaxy, where hydrogen is not present.^17,21^ Schmidt *et al.* discovered the (2 × 2)-2D-2H surface to be dominant in their calculated surface phase diagram when including hydrogen in the surface reconstruction and thus likely to occur under a variety of preparation conditions given the presence of hydrogen.^17^ Their surface modeling has been verified and scrutinized in various studies and displays a characteristic (2 × 1) pattern in the LEED image, with streaks at half order in the ×1 direction.^12,22^ (ii) The second possible InP(100) surface reconstruction is the (2 × 4) mixed dimer surface, which is terminated by P–In mixed dimers, also called the “In-rich” surface.^23,24^ While the (2 × 4) In-rich surface states have been extensively investigated,^23,25−27^ the understanding of the (2 × 1)-like P-rich InP(100) surface is still limited.^4,15,17^ Töben *et al.*(27) measured time-resolved two-photon photoemission spectroscopy (tr-2PPE) on P-rich, p-doped InP(100) but were unable to clearly resolve surface states, though they were able to identify states on the In-rich, n-doped InP(100) surface. They were also unable to resolve higher-energy conduction band states due to limited pump photon energies.^27^

Although p-InP(100) photoabsorbers show promising performance in PEC,^3,7−9^ a better understanding of the underlying carrier dynamics and electronic pathways is crucial for further improving their performance as photoelectrodes. Notably, for p-InP photocathodes, the highest PEC efficiency has been achieved by incorporating a 10 nm TiO_2_ protective layer.^8^ This configuration can yield a total photovoltage of 785 mV, providing a significant portion of the minimum required photovoltage of 1.23 V for photoelectrochemical water splitting.^3^ Other common surface modifications include PEC passivation by repeated surface oxidation and reduction,^28^ formation of a front surface field to enhance contact selectivity,^29,30^ as well as deposition of hydrogen evolution catalysts such as Pt,^31^ which have been found to significantly increase PEC performance. Understanding the underlying InP photoabsorber and its interface to such functional surface layers is crucial in improving device performance.^28,31^

InP(100) has also attracted attention due to its potential use in hot-carrier solar cells, making use of phonon-bottlenecking to increase hot exciton lifetimes. Phonon bottlenecking arises from the presence of discrete energy levels, usually found in semiconductor nanocrystals or quantum dots, spaced in the order of hundreds of meV. As a result of these states being discrete rather than a continuous density of states (DOS), limited numbers of phonons are available for nonradiative thermalization of excitons. As a large number of phonons are required at higher densities of excited electrons, thermalization slows above a critical injection carrier density.^32−34^ Literature suggests the possibility of a phonon bottleneck in InP,^35,36^ which Clady *et al.* first reported experimentally in 2011.^37^ A recent study by Zhang *et al.* reviewed the mechanisms for phonon-bottlenecking in bulk-InP and InP nanostructures.^36^ Understanding thermalization at the InP surface both in identifying surface states and determining their lifetimes is an important step in elucidating the suitability of InP for hot-carrier solar cells. In addition, InP is also an important material in other applications, such as laser diodes and photonic integrated circuits, and its use in these as well as other optoelectronic devices^38,39^ is an active research topic. For example, recent work by Proppe *et al.* explored the use of colloidal p-type, P-doped indium phosphide (100) quantum dots as a single-photon source for application in quantum photonic techniques.^40^

In our investigation, we focused on the P-rich p(2 × 2)/c(4 × 2)-reconstructed InP(100) surface prepared by MOVPE. To benchmark the surface composition, X-ray and ultraviolet photoelectron spectroscopy (XPS, UPS), reflection anisotropy spectroscopy (RAS), LEED, and atomic-force microscopy imaging techniques were employed. To study normally occupied and, particularly, unoccupied electronic states around the band gap, we used tr-2PPE. While single photon emission methods like XPS/UPS do not provide access to unoccupied states, tr-2PPE allows for their observation, including lifetime information.^41−43^ The short pump and probe pulses (30–40 fs fwhm) provide sufficient time resolution to observe scattering events at both near-surface and surface states.^16,21^ Moreover, the relatively high photon energies of the pump pulse also allowed us to probe for the first time higher-energy states in the p-InP conduction band.

## Methods

### Sample Preparation in MOVPE

P-rich (2 × 1)-like InP(100) surfaces were prepared in a horizontal-flow MOVPE reactor (Aixtron, AIX-200) on p-doped InP(100) substrates with a doping concentration of 2 × 10^18^ cm^–3^ (Zn-doped) and a 0.1° miscut toward the [111] direction. Prior to the surface preparation, the InP(100) wafers were deoxidized at 620 °C for 10 min with a supply of tertbutylphospine (TBP) precursor, using H_2_ as a carrier gas. Subsequently, at 600 °C, a 100 nm thick homoepitaxial layer was grown with supply of TBP, trimethylindium (TMIn), and diethylzinc (DEZn) as the p-dopant precursor. After the homoepitaxial growth, the samples were cooled under TBP and a phosphorus dimerized surface was prepared.^44,45^ Throughout the entire process, including the surface preparation, changes to the atomic order at the surface were monitored *in situ* with RAS, an optical technique particularly sensitive to the asymmetrically reconstructed (100) surfaces of cubic crystals.^46^ RAS (LayTec EpiRAS-200) was aligned such that the difference in reflection along [01®1] and [011] was measured. The as-prepared P-rich InP(100) samples were transferred from the MOVPE reactor to ultrahigh vacuum (UHV)-based surface characterization techniques in a UHV shuttle with a base pressure of ≤5 × 10^–10^ mbar.^47^ Prior to the 2PPE measurements, the surface symmetry and the chemical composition of the samples were measured by LEED (Specs ErLEED 100-A) and XPS (Specs Focus 500 and Phoibos 150), respectively.

### Time-Resolved Two-Photon Photoemission Spectroscopy

**Figure 1:**
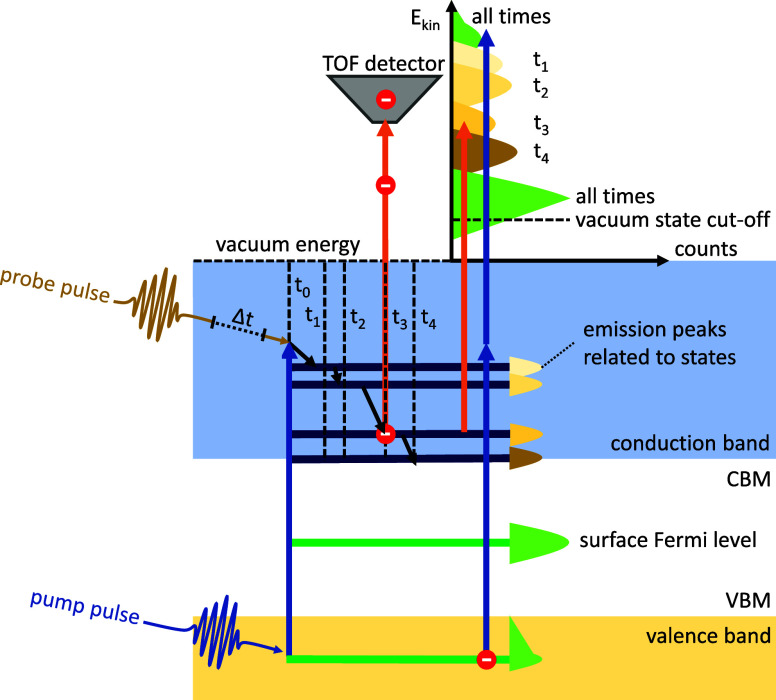
Schematic of the tr-2PPE emission process. Electrons are photoexcited by a pump pulse and then emitted after a time delay Δ*t* by a probe pulse, after which their kinetic energy is measured in a time-of-flight detector. This leads to emission peaks correlating to electronic states in the material at time delays corresponding to the maximum occupation of the respective state.

In tr-2PPE, electrons are photoexcited within the material by using a pump pulse, followed by their thermalization and settling into normally unoccupied states inside or above the band gap. Subsequently, these electrons are photoemitted by a probe pulse, which has lower intensity than the pump pulse and can be delayed in time with respect to the pump pulse (Figure S1 in the Supporting Information). The energy distribution and timing of the photoemitted electrons provide insights into the relative energies and lifetimes of states within the sample material. Emission peaks are observed at kinetic energies correlating to electron states in the material, and their decay shows emptying of the corresponding state. By scanning the time delay in small steps, such as 5 fs, and plotting the kinetic energy of the photoemitted electrons, the electron dynamics in the near-surface region can be visualized (Figure 1). Both occupied and unoccupied states can be observed; the unoccupied conduction band states are of particular interest, since they are not accessible through XPS or UPS.^27,41,42^ Literature values for the inelastic mean free path of electrons in InP are given at 5 Å at 50 eV, increasing to above 25 Å at lower energies, though no precise values have been reported.^48^ This agrees well with values from previous studies, in which some of the present authors estimated the information depth of tr-2PPE to be approximately 45 Å in n-type InP^27^ for photon energies at 4.66 eV, allowing for the observation of both surface and bulk features.

In tr-2PPE experiments, different photon energies can be utilized for the pump and probe pulses. This allows electrons to be excited to a range of normally unoccupied energy levels. Note that a difference between pump and probe energies will lead to an asymmetry of the tr-2PPE data along the time axis. The order of the pump and probe photons can determine which unoccupied states are accessible. This also impacts the thermalization kinetics, and both of these factors affect the kinetic energy of the photoemitted electrons. The experimental setup is outlined in Notes S1 and S2 in the Supporting Information.

Density functional theory (DFT) calculations are performed in order to assist in the interpretation of the measurements. Here, the Vienna Ab initio simulation package^49^ is used. The electron exchange and correlation is described within the general gradient approximation using the PBE functional.^50^ The electron–ion interaction is described by the projector-augmented wave method in a plane wave basis.^51,52^ The surface is modeled by a long slab consisting of 60 atomic layers in order to reduce quantum confinement effects.^53^ This rather large slab provides sufficient material to model both bulk and surface states realistically. A vacuum region of about 44 Å separates the material slabs in the surface in the normal direction. The slab bottom layer is indium-terminated and passivated with artificial hydrogen atoms (*Z* = 1.25). A surface dipole correction^54^ is employed in order to reduce spurious effects due to the nonequivalent slab surfaces. The wave functions are expanded in a plane wave basis with an energy cutoff of 500 eV. The surface is relaxed until the forces are smaller than 0.02 eV/Å. A 4 × 4 *k*-point mesh is used for the sampling of the surface Brillouin zone. The electronic DOS is sampled in a small region in the Brillouin zone center. The scissors operator approach is used to account for self-energy effects in the calculated excited-state energies. The scissors shift is obtained by energy alignment to the hybrid DFT results. The present hybrid DFT calculations on InP bulk using the HSE functional^55^ predict a band gap of 1.35 eV, in excellent agreement with the measured value. Finally, we use the Slater–Janak technique^56^ to calculate the energy position of the charge-transition level arising from surface H vacancies that pin the Fermi level at defective surfaces.^57^

## Results and Discussion

**Figure 2:**
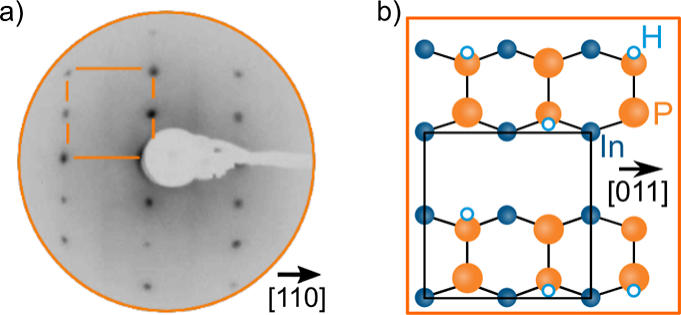
(a) LEED of the P-terminated InP(100) p(2 × 2)/c(4 × 2) surface reconstruction and (b) schematic structure of this surface reconstruction. The black square shows a p(2 × 2) unit cell, in which the adjacent P–P dimers rows are arranged in-phase. The c(4 × 2) unit cell is not indicated here.

LEED of the surface was performed to verify the surface reconstruction after transfer in UHV. Figure 2a shows the LEED pattern of the P-rich InP(100) surface with clearly visible (2 × 1) diffraction spots, as well as diffused streaks along the [01̅1] direction, a signature for this surface reconstruction prepared in MOVPE ambience.^13,15,17^ Random occupation of hydrogen within a surface unit cell leads to the formation of diffuse streaks in the LEED pattern. This occurs through the superposition of unit cells with varying translation periodicities such as p(2 × 2)/c(4 × 2). Figure 2b shows the corresponding model of the P-rich, p(2 × 2)/c(4 × 2) surface reconstruction. Further details on the verification of the surface cleanliness and reconstruction are given in Note S3 as well as Figures S3 and S4.

**Figure 3:**
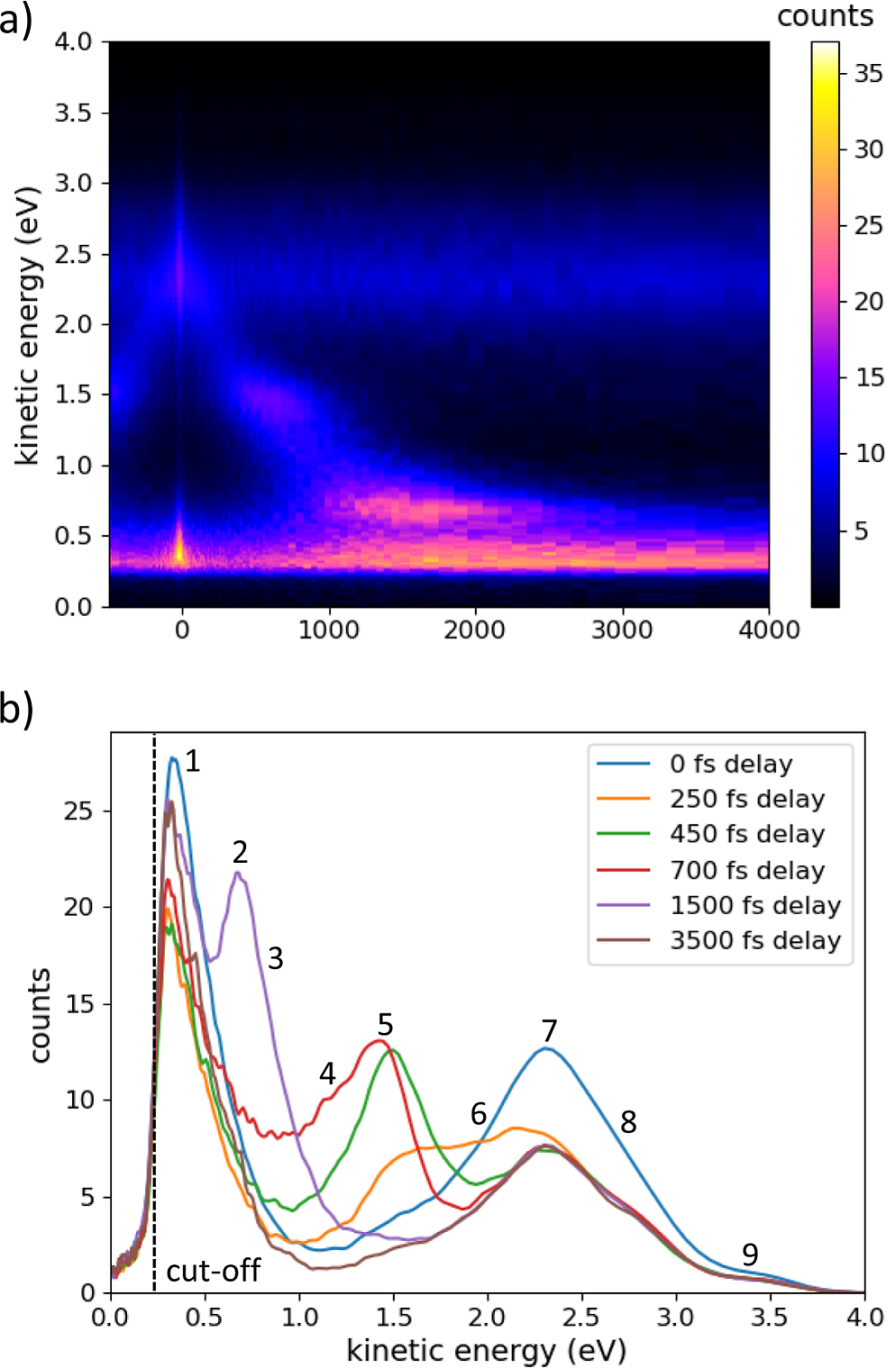
(a) Tr-2PPE transient of P-rich InP(100). Counts of photoemitted electrons are given as a function of delay time between pump and probe and kinetic energy of emitted electrons. The yellow and pink areas denote combinations of delay and energy where a larger number of photoemitted electrons were detected, and the blue and black areas had little to no emission. (b) Corresponding spectra showing the energetic positions of detected states (number from 1 to 9), with delay times chosen such that the apparent features in the tr-2PPE transient are shown in detail.

**Figure 4:**
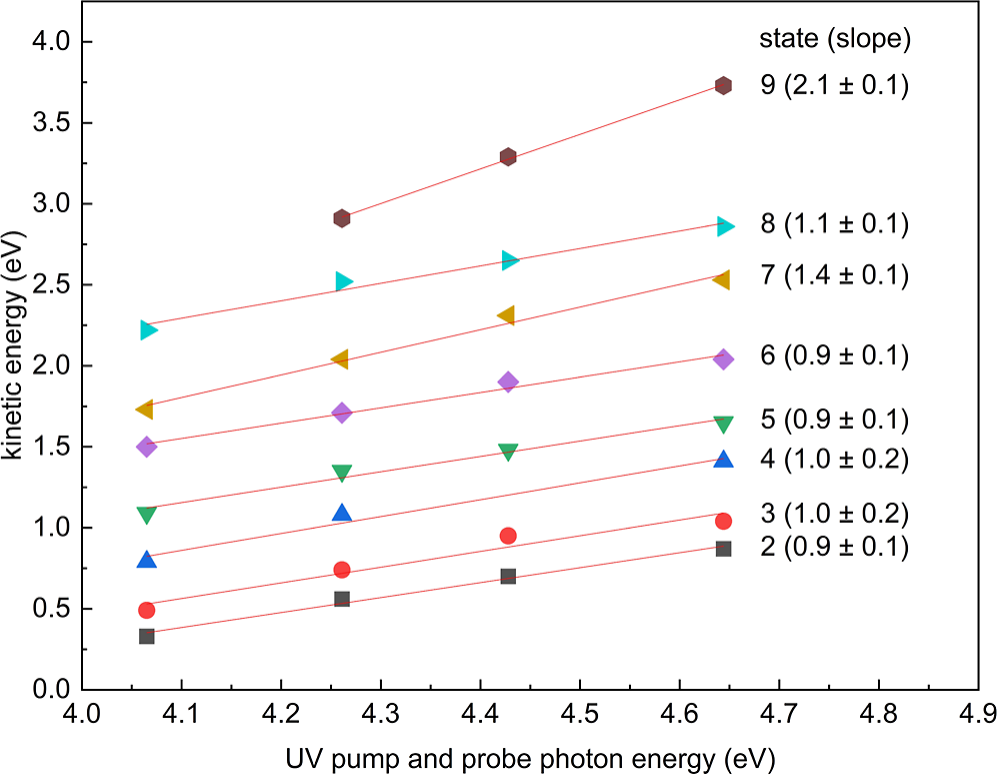
Positions of peaks are found in Figure 3 as a function of pump and probe energy. State 1 is partially or fully cropped in several measurements and was therefore not included. States 2–8 show clear linear shifts with photon energies. Gradients are given next to each fitted line on the right and are used to identify states as occupied or unoccupied.

Figure 3a shows tr-2PPE measurements from the P-rich InP(100) surface using a 276 nm UV pump and probe beams at a 2:1 intensity ratio, displayed in a pseudo-3D transient plot. The detected kinetic energy of emitted electrons is plotted against the time delay between the pump and probe pulses. The color scale indicates the electron counts at any given combination of delay and detected kinetic energy. Positive delay times denote the pump following the probe, and at negative delay times, the probe leads the pump. Due to the identical pump and probe energies in this measurement, this results in a symmetry around delay = 0 fs. To avoid redundancy, only data at positive time delays are shown. Figure 3b shows kinetic energy spectra extracted from Figure 3a, in which emission peaks can clearly be observed at a range of time delays. More detailed spectra with a larger number of delay slices are given in the Supporting Information (Figure S5). Varying photon energies as shown in Figure 4 (described below) reveals that the lowest energy peak visible in Figure 3b (denoted by number 1) is cropped and not fully resolved. This suggests the presence of vacuum states that prevent the emission of electrons with kinetic energies below a cutoff value of ∼0.23 eV *vs* vacuum (estimated from the half-maximum of the peak).

In addition to the partially cropped lowest energy state that we will refer to as state 1, eight other states are observed at kinetic energies of 0.69, 0.84, 1.21, 1.48, 1.93, 2.33, 2.77, and 3.40 eV (states 2–9). States 1, 2, 5, and 7 can be distinguished in the 3D transient plot in Figure 3a, but states 3, 4, 6, 8, and 9 can only be clearly identified in the corresponding spectra shown in Figure 3b. There is an uncertainty in state positions of about 0.05 eV due to the uncertainty in UV photon energies, corresponding to the uncertainty in the photon wavelength of about 3 nm. State 7 at 2.33 eV is fitted as a double peak feature with peaks 0.1–0.2 eV apart in several measurements, although the relative proximity and width of these peaks make fitting them as separate peaks unreliable. There further appears to be a peak between state 6 and 7, though it is only observed around 200–300 fs delay in some samples, and not clearly observed in others. To enhance peak clarity and verify fitted peak positions, additional peak deconvolution methods were employed; this is given in more detail in the Supporting Information. The steady-state background is subtracted from spectra to enhance clarity of time-dependent features (Figure S6); the sum of gradients of the delay slices are plotted in Figure S7 both with and without background. Principal component analysis is performed on the data after subtraction of the background (from Figure S6); this is given in Figure S8 and Note S4.

To extract the spectra in Figure 3b, we averaged tr-2PPE measurements over 3–5 adjacent delay steps at several time delay values. Temporal positions for averaging were selected to clearly visualize the major features of the transient plot in a cross-section, in particular, the kinetic energies of observed electronic states. To enhance the data presentation, we binned the data and applied Gaussian smoothing to average the data over small fluctuations between measurement points. Note that a bias voltage of +0.2 eV was applied between the electron detector and the sample during different measurements; this value was subtracted from the kinetic energies of electrons in the presented data. Multiple peak Voigt fits were utilized to determine the position of each peak. Voigt fitting was chosen, as it accounts for both the Lorentzian components to the energy distribution, caused by the uncertainty principle, as well as contributions by the Gaussian character of photon energy distributions in the beams, phonon broadening and influences by the experimental setup^58^ (Note S5). Example fits are given in the Supporting Information (Figures S9 and S10); a penalty for proximity between was applied to reduce the likelihood of assigning peaks into the flanks of other peaks where not necessary.

To distinguish between occupied and unoccupied electronic states, we use the specific slope of kinetic energy against photon energy, as shown in Figure 4, for four different UV energies (4.07, 4.26, 4.43, and 4.64 eV). In this analysis, the UV beam was split to provide the same wavelength for both the pump and probe, as shown in Figure 3. For electrons directly emitted from an occupied state, the kinetic energy scales with twice the photon energy due to the two-photon emission process. This could be a bulk or surface valence band state, surface defect states in the band gap, or a surface state pinning the Fermi level. On the other hand, if a photoexcited electron thermalizes into an unoccupied state and is subsequently emitted by the probe pulse, the resulting change in kinetic energy of the emitted electron scales with 1x the photon energy. This principle is outlined in Figure S11 and Note S6.

By observing these distinct scaling relationships between the kinetic energy and photon energies for different UV energies (Figure 4), we can effectively discern and characterize the electronic states of the material. For each electronic state analyzed, a strong linear correlation is observed between the photon energy used in both pump and probe beams to the kinetic energies of the photoemitted electrons. High R-square values (≥0.95 for all states) signify the robustness and accuracy of this correlation. Note that we did not include State 1 in this analysis; the low kinetic energy of this state makes it challenging to precisely track it over the entire range of photon energies.

He I UPS analysis of the surface indicates a work function of 4.80 eV, taken as the half-maximum of the lower edge of emission in UPS (Figure S12). The work function as measured by UPS describes the energetic difference between the Fermi level and the lowest energy emitted electrons, the latter being the lower energy cutoff observed in Figure 3. The position of the Fermi level relative to vacuum *E*_F_ is related to the work function Φ and the upper edge of vacuum states *E*_vac_ by
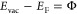


Given measured vacuum states up to 0.23 eV as described earlier, and the 4.80 eV work function, we calculate the Fermi level to be 4.57 eV below the vacuum level. A recent study by Moritz *et al.*(15) on the p(2 × 2)/c(4 × 2)-reconstructed P-terminated InP(100) surface reported Fermi-level pinning due to surface defects, correlating to the UPS work function. This was corroborated in a recent DFT paper by some of the present authors.^59^ The work function is 0.31 eV larger than the 4.49 eV photon energy used in the tr-2PPE measurements and should therefore only allow for 2PPE from occupied states and no single photon emission (1PPE). Due to the uncertainty in pulse energies, some electrons may also be emitted in 1PPE from the surface Fermi level, leading to the partially cropped state 1.

Based on the discussion mentioned above, we can assign the individual states as follows:

1 State 1 is associated with a surface state that pins the Fermi level. This state has been reported in the literature,^15^ where it was assigned to partially filled P dangling bonds on the surface resulting from H-vacancies.^59,60^ Due to its low kinetic energy and the presence of vacuum states, precise peak maximum identification and tracking is challenging.^60^
2 States 2, 3, 4, 5, 6, and 8 exhibit a scaling factor of 1.0 ± 0.2 between photon and electron energy, suggesting that they are intermediate, normally unoccupied states populated with electrons pumped from filled valence band states.
3 State 7 shows an intermediate scaling factor of 1.4 ± 0.1. This indicates a more complex behavior and potentially a different underlying mechanism.
4 State 9 has a scaling factor of 2.1 ± 0.1, indicating a normally occupied state.

A summary of these results is given in the Supporting Information (Table S1).

**Figure 5:**
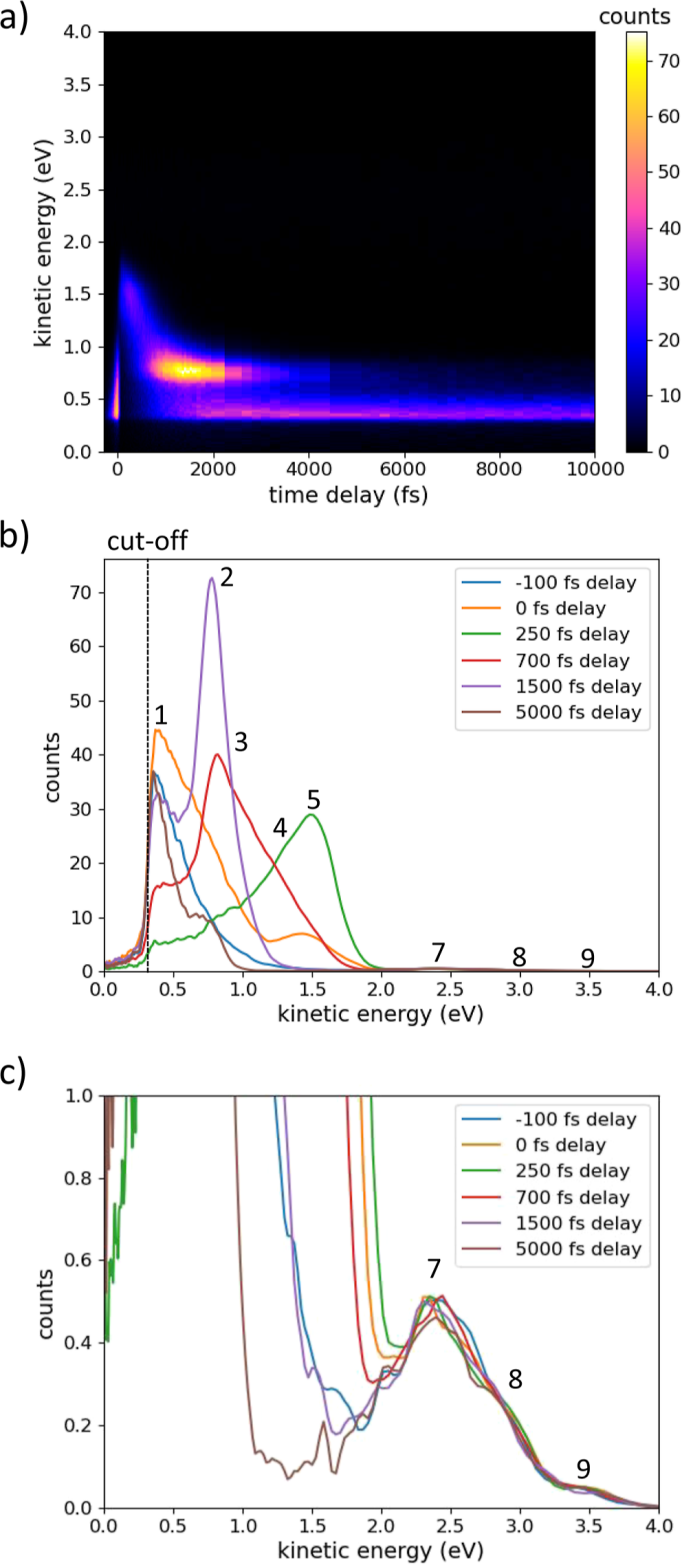
Tr-2PPE transient of P-rich InP(100) surface using 533 nm (2.33 eV) pump and 276 nm (4.49 eV) probe in (a). For positive delay values, the pump is followed by the probe, and for negative delay values, it is the reverse. The corresponding spectra are shown in (b), with a zoomed in region near states 7–9 in (c).

In the subsequent tr-2PPE experiments, a 533 nm (2.33 eV) VIS pump is used, while keeping the probe at 276 nm (4.49 eV), maintaining a 50:1 intensity ratio between the pump and probe. Hereafter, we refer to the measurements with the 533 nm pump as VIS–UV and those using the 276 nm pump as UV–UV. Figure 5 displays the 3D-transient and kinetic energy spectra obtained from these measurements. At negative delay values, the VIS beam acts as the probe and the UV beam as the pump. Here, the signal decays within 1 ps, likely as electrons thermalize to energy levels the VIS beam no longer has the necessary photon energy to emit from. The dynamics at positive delay values show clearly identifiable electronic states at kinetic energies of 0.78, 1.02, 1.26, and 1.50 eV and weakly observed states at 2.32, 2.78, and 3.49 eV. These values correspond to the previously observed states 2–5 as well as 7–9 in the UV–UV experiment (Figure 3), while no clear VIS–UV signal was observed for state 6. The half-maximum of the lower energy cutoff is located at 0.23 eV (as in the UV–UV excitation scheme). It should be noted that the lower energy cutoff in tr-2PPE varies between measurements in a range of up to 0.25 eV, with most values within ±0.05 eV of values reported here. This may in part be due to surface inhomogeneities depending on which position on the sample is chosen, although the larger contributor is likely to be surface charging. The downward band bending at the surface due to Fermi level pinning may lead to charge build up from photoexcited electrons migrating to the surface, which would create a barrier to emission of electrons, thereby changing the observed cutoff energy. As outlined by Lüth,^61^ for III–V semiconductors such as InP, surface band bending is pinned for surface state densities above a critical point at around 10^12^ cm^–2^. Larger surface state densities do not shift band bending significantly, with changes on the order of tens of meV or below, provided the minimum state density required for pinning of the Fermi level is not crossed. In our experiments, we avoided this by limiting pump and probe intensities, such that below the maximum intensity, there is no strong intensity dependence of the cutoff or peak positions. For the case of UV–UV, we found a doubling of the intensity to result in a shift in band bending of around 0.03 eV.

It should be noted that VIS–UV (Figure 4) cannot access all the states that UV–UV (Figure 3) does due to the lower pump energy. However, there are still some UV—pumped electrons present in the VIS–UV measurements, as some electrons are pumped by the probe beam and are subsequently emitted by photons from the same probe pulse. The signal from these UV—pumped electrons will, however, be relatively weak in the VIS–UV measurements due to the 50:1 difference in pump and probe beam intensities, and it will not vary with the delay time. The VIS–UV signals from states 6–9 are about 2 orders of magnitude smaller in intensity than the other states, indicating that they are energetically inaccessible to the 2.33 eV VIS pump. In contrast, states 1–5 show clear signals for both the VIS–UV (Figure 5) and UV–UV (Figure 3) excitation schemes.

Due to its 2x scaling with photon energies, state 9 is likely a valence band state, hence labeled as V1. The energy level *vs* vacuum of an occupied state *E*_occ._ in UV–UV is obtained by subtracting twice the UV photon energy *h*ν from the measured kinetic energy *E*_kin_
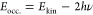


This results in an energy of −5.55 eV *vs* vacuum for V1. The valence band maximum (VBM) is determined *via* linear fit of the higher-energy flank of V1 and found to be at 3.84 ± 0.05 eV kinetic energy, which corresponds to −5.11 ± 0.05 eV relative to the vacuum energy (Figure 9 and Table S1). Schmidt *et al.* predicted valence band states 0.1 and 0.4 eV below the VBM in In-rich n-InP(100), the former associated with In-features not observed in the surface reconstruction described here.^23^ The valence band state 0.4 eV below the VBM in their calculations was associated with dangling bonds at the top P atom, which likely correlates with the valence band state V1 observed here.

**Figure 6:**
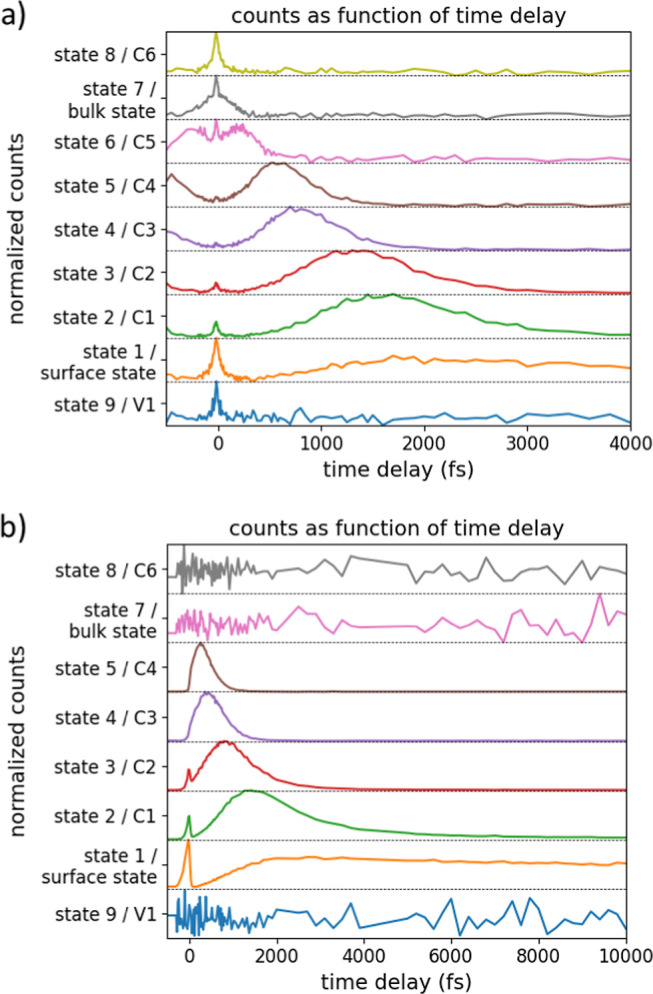
Emission counts per second from the observed states in UV–UV from Figure 3 in (a) and VIS–UV from Figure 5 in (b) as a function of time delay. The total counts in the region of ±0.05 eV around the peak energies is plotted. States are identified by their number according to Figures 3, 5, and Table S1.

**Figure 7:**
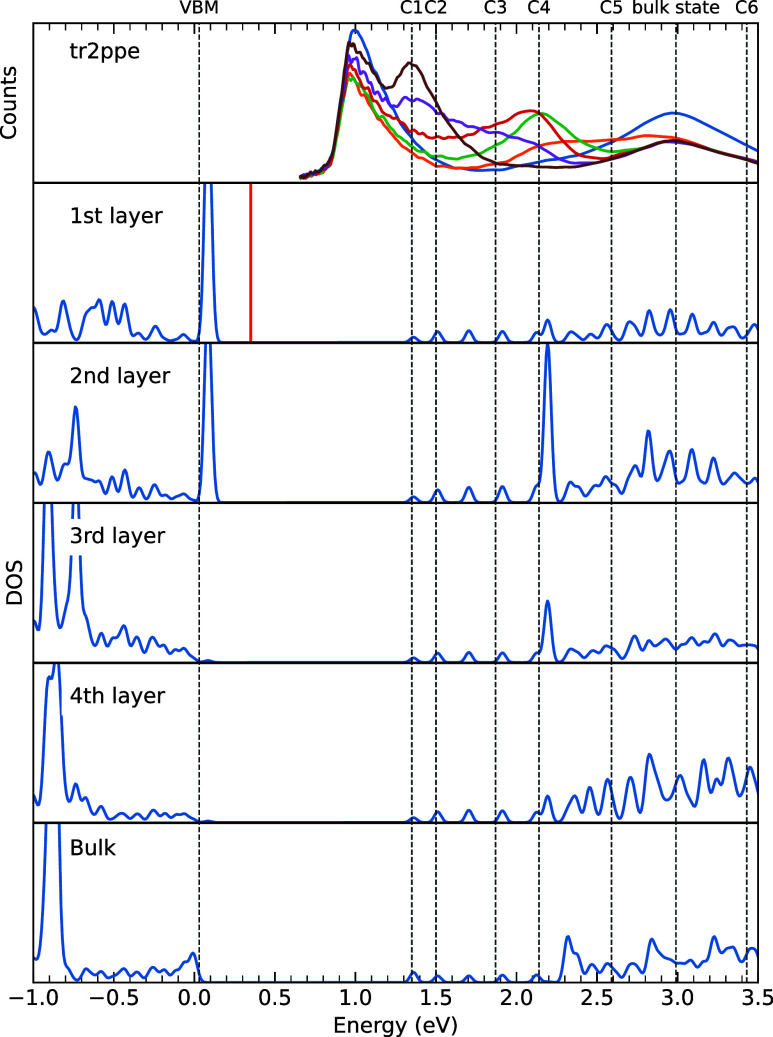
DFT-calculated DOS for the uppermost four atomic layers, as well as for the bulk material in comparison to the tr-2PPE data (taken from Figure 3b). The red vertical line indicates the calculated defect energy level. The dashed lines are the energy levels of the electronic states determined from the tr-2PPE measurements. The bulk state denotes a cluster of states identified as a bulk-to-surface transition, state 7 previously.

**Figure 8:**
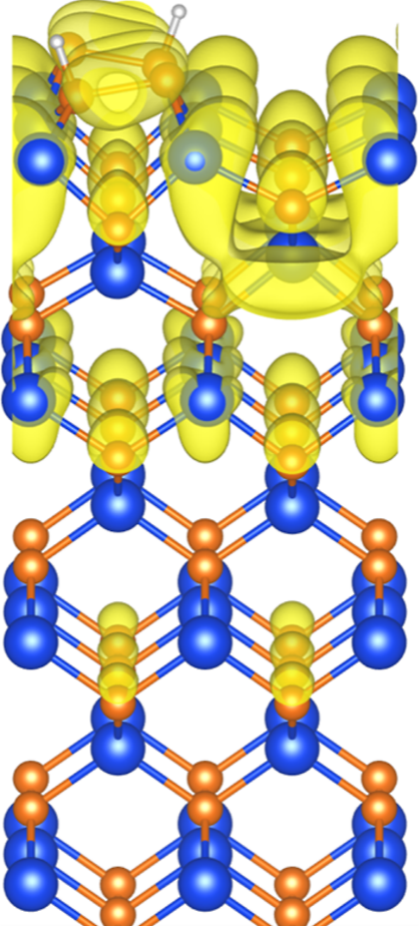
Calculated orbital character of state assigned to C4 in Figure 6. Blue, orange, and gray spheres represent the In, P, and H atoms, respectively.

**Figure 9:**
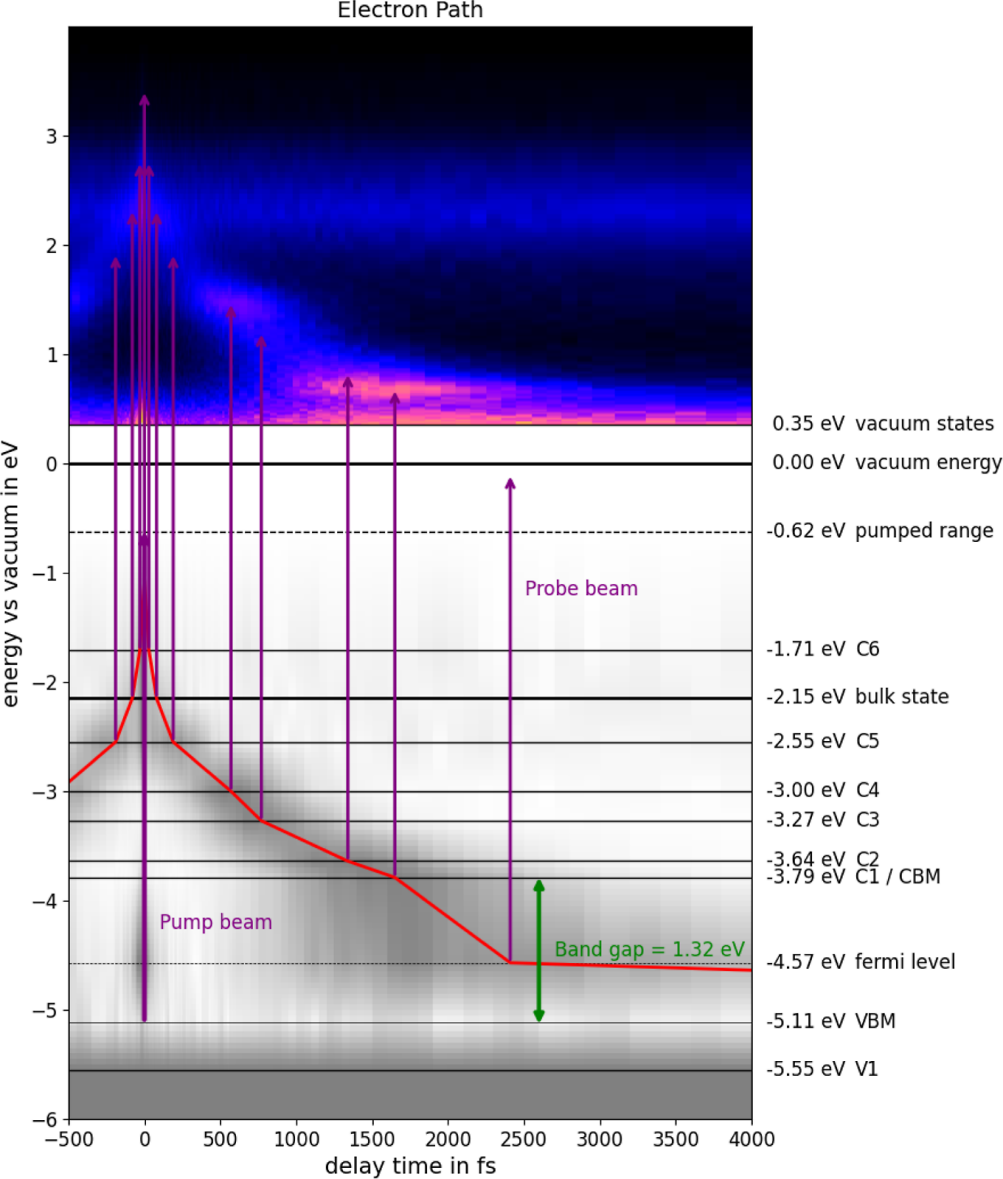
Energy levels in the pure P-rich InP surface are given relative to the vacuum energy. Electron relaxation pathways are shown for electrons photoexcited by UV pump; the band gap of 1.32 ± 0.08 eV is indicated in green.

Previous DFT calculations^15^ suggested that the bulk Fermi level state should be located 0.04 eV above the VBM. The surface state pinning the Fermi level at −4.57 eV *vs* vacuum, as previously outlined, is energetically 0.54 eV above the VBM, indicating an amount of surface band bending of 0.50 ± 0.10 eV. This observation is confirmed in similar measurements on another sample, which indicated a work function of 4.69 eV, corresponding to a Fermi level position of −4.47 ± 0.05 eV *vs* vacuum. In tr-2PPE, a VBM of −4.93 ± 0.05 eV was measured on that sample, resulting in 0.42 ± 0.10 eV of band bending, compared to 0.56 eV band bending reported for the P-rich InP surface in literature.^15^ States 2, 3, 4, 5, 6, and 8 were determined to be unoccupied states, as described previously, and are therefore labeled as conduction band states C1–C6. Their positions *E*_unocc._*vs* vacuum are obtained by subtracting the UV probe energy *h*ν from the measured kinetic energies *E*_kin_ (see Figure 9) as
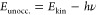


The results are given in Table S1 (and are summarized at the end of this paper, in Figure 9). The emission peaks related to C1 and C2 overlap each other, as do the peaks corresponding to C3 and C4. At lower time or energy resolution, they may therefore appear as two states C1/2 and C3/4. Literature on the In-rich n-InP surface reports what is described as two surface bands, one around C1/2 as well as one around C3/4, which are attributed to surface resonances.^26,62^ C5 and C6 are not observed, possibly due to their smaller emission amplitudes.

To determine the surface band gap, usually the VBM and conduction band minimum (CBM) positions are found by extrapolation of the valence and conduction band DOS to zero toward the band gap. The band gap is then the difference between the VBM and the CBM. As we observe discrete conduction band states instead of a continuous conduction band DOS, we take the surface band gap to be between the VBM and C1 instead. The valence band does not have the same clear separation of states and may be more closely represented by a continuous DOS, possibly with strong bulk or subsurface contributions. This would be worth further exploring, *e.g.*, by determining the stability of V1 under surface modification. The band gap is determined as 1.32 ± 0.08 eV, consistent with InP band gap values in literature, which reports InP band gaps ranging from 1.34 to 1.35 eV.^8,13,63^

State 7 scales with photon energy at a factor of 1.4, clearly consistent with neither an occupied nor an unoccupied state. DFT DOS calculations of the first four atomic layers, as shown below in Figure 7, assist in the assignment and discrimination of bound surface states, surface resonances, and surface modified bulk states (see also Figure 8). The energetic overlap and energetic degeneracy as well as the assumed microscopic proximity of the surface and bulk-like states point to efficient electron scattering between them. This may explain the 1.4x scaling of state 7 with photon energy, indicating a bulk-to-surface transition. Tr-2PPE experiments on In-rich, n-InP by Jodi *et al.*(62) and Töben *et al.*(26) describe a state at almost the same energetic position as our state 7. Töben speculated on the cause of this state as a volume transition between bulk and surface and observed a 1.32 ± 0.11 scaling of emission energy with pump photon energy, consistent with our findings.^64^ Almost no change in the 2PPE signal of this state under hydrogen exposure was found by Töben *et al.*,^26^ which supports the original assumption of electron transfer from the bulk toward the surface. In contrast, we found other surface states to be suppressed for samples exposed to atmospheric conditions or water exposure, which we describe in more detail in a follow-up paper.

### State Occupancy as a Function of Time Delay

In Figure 6a,b, we present the total emission counts in a 0.1 eV energy window around each state as a function of delay time for the previously observed electron states in UV–UV and VIS–UV, respectively (Figures 3 and 5); data are taken within ±0.05 eV of each state. Emission counts for the surface state pinning the Fermi level are taken just above the energy drop-off at 0.35 eV, which likely only represents the upper-energy flank of this peak. Nonetheless, it should provide a good approximation of the time-dependent occupation. From Figure 6a (UV–UV scheme), the behavior of the different states is as follows:

1 V1 (valence band state): V1 exhibits a sharp emission peak at 0 fs delay time, with no other time dependence. This suggests that electrons from V1 are directly emitted without any significant temporal dynamics.
2 Surface state that pins the Fermi level: the occupancy of this state displays a 0 fs delay peak attributed to direct 2PPE, followed by a gradual increase as it is filled by thermalization of electrons from conduction band states.
3 Electron thermalization: after initial excitation, electrons in UV–UV undergo thermalization through the following conduction band states in sequential order: C6, bulk state, C5, C4, C3, C2, and C1 and eventually return to the Fermi level on average 1.8 ± 0.2 ps after initial excitation.

In Figure 6b, the VIS–UV measurement exhibits the same thermalization path as that observed in Figure 6a. In this case, electrons are photoexcited from the VBM at −5.11 eV *vs* vacuum by the VIS pump to 0.22 eV above C4, but 0.23 eV below C5. From there, electrons feed into C4, C3, C2, and then into C1 and finally return to the surface state pinned Fermi level. Notably, C5, the bulk state, and C6 are not energetically accessible to the VIS pump and can only be accessed by photons from the UV pump. Consequently, there is no time development for these states in the VIS–UV measurement. C5 (= state 6, see Table S1) is not listed, as it was not clearly observed in Figure 5b,c.

The lifetimes of conduction band states C1–C5 are determined by fitting an exponential decay function into the declining flank, starting at the half-maximum height and going toward increasing delay values. In UV–UV, this gives decay time constants for C1–C5 of 760, 520, 270, 250, and 130 fs, respectively. The VIS–UV measurement gives decay time constants for C1–C4 of 1550, 650, 290, and 240 fs, respectively. Different electron decay paths will lead to states both being filled and emptying simultaneously, leading to increased measured lifetimes, as well as differences due to differences in electron–electron and electron–phonon scattering with pump intensities and -energies.^41^ There are also possible effects of phonon-bottlenecking due to differences in pump and probe intensities, which is known to occur in bulk InP.^36,37^ Diffusion of carriers due to surface band bending may add on the order of 15 fs to lifetimes; a brief calculation is given in Note S7. This also indicates that excited carriers reach the surface well before thermalizing to the band edges, a crucial basis for the design of hot-carrier cells.

In order to assist the interpretation of the experimental data, DFT is used to calculate the atomic-layer resolved electronic DOS; see Note S8. Figure 7 shows the DFT-calculated DOS, sampled around the Brillouin zone center (blue curves). The vertical red line indicates the calculated energy of the charge-transition level assumed to pin the Fermi level on P-rich InP (001):H surfaces. This transition level, calculated to be at 0.37 eV *vs* the VBM, is related to P dangling bond defects resulting from H desorption.^15,59^ It is slightly below the measured Fermi energy at 0.54 eV *vs* the VBM, which might be due to limitations of the present theory as well as due to additional surface defects not considered in the calculations. As the tr-2PPE data are cut off at the lower end of the energy range due to vacuum states, the spectra do not show the surface state directly; experimental confirmation was performed in XPS as described above.

Starting at the upper edge of the energy range, we observe that C6 correlates to DOS peaks in the fourth layer and below in the bulk states. The state we interpreted as a bulk-to-surface transition (state 7 previously) appears to be made up of several DOS peaks between the second and fourth layers, with a pair of states in its center in the first and second layer. The double peak feature was noted previously, although we had not been able to fit this feature reliably. The DOS indicates states correlating to this peak in all calculated layers, lining up well energetically, which would allow electrons to move between the bulk and surface. This supports the interpretation of the data in terms of bulk-to-surface transitions.

Given that the bulk-to-surface transition appears to consist of a cluster of states rather than a single state, we can understand the perceived 1.4x scaling in emission energy with the photon energy described above. The left flank of the bulk-to-surface transition is set by the energetic position of the associated unoccupied conduction band states at this position, which scales with 1 × photon energy. The upper-energy flank of the bulk-to-surface transition is likely not caused by the conduction band DOS, but rather by the valence band DOS. This is projected onto the available conduction band states, determining the bulk-to-surface transition shape from its maximum to its uppermost edge in emission. This flank will then scale with a 2 × photon energy. The peak position of the bulk-to-surface transition is the overlap of the gradients from the left and right flank, scaling at 1 and 2 × photon energy, respectively. The projection of the valence band edge onto the DOS of the bulk-to-surface states will therefore cause the resulting peak to scale at around 1.5 × photon energy as observed.

This cluster of states in the near-surface region ensures that for any electron within it, there are several available states within about 100 meV energetically in the adjacent atomic layers. As these states are spatially as well as energetically close to each other and span a range of more than 1 eV, they provide a quasicontinuous band, where electrons may easily transition between states. The denser clustering of states in this region as compared to the more distinct conduction band states C1–6 will facilitate easier electron tunneling or hopping between states, as state wave functions likely overlap. When photoexcited electrons diffuse toward the surface due to the downward band bending as described above, this cluster then lowers the energy barriers to electron movement, enhancing the transport of electrons within this range. This leads to the label of this dense cluster of states as a bulk-to-surface transition, which acts as a reservoir of electrons, which can be efficiently transported to the surface, explaining enhanced emission at these energies as observed experimentally (Figure 3). Further, the clustering of states may lead to a redistribution of electrons without significant loss of energy to phonons; this may explain the more “smoothed out” appearance of this feature as compared to the other, more distinct conduction band states.

C5 appears to be mainly caused by features in the uppermost four atomic layers and is therefore likely filled through electrons photoexcited in the bulk, moving through the bulk-to-surface transition state as previously suggested. There appears to be features between the bulk-to-surface transition and C5, particularly a state in the second layer. This likely correlates to the weakly observed feature around 250 ± 50 fs noted earlier, though that could not be fitted reliably. As electrons reach the surface from the bulk and transition into C5, this state appears to be briefly occupied for some tens of fs. From the calculations, C4 is related to features in the upper four atomic layers from DOS with a low bulk contribution. The DFT calculations show that the strongest contributions to C4 are related to In–P σ-bonding states between the first and second as well as second and third atomic layer. In addition, we have contributions from P-dimer localized π bonding states as well as bulk states; see Figure 8. C4, C2, and C1 appear to be related to both bulk states as well as surface resonances, which again would allow for efficient migration of electrons from bulk to surface.

In addition to the bulk-to-surface transition identified in the experimental results, we therefore have two other likely points at which electrons may migrate to the surface, at C4 and C2/1. Clady *et al.* showed, using time-resolved photoluminescence, energetic transitions across the band gap of 1.70, 1.88, and 2.40 eV, allowing access to the Γ, *L,* and *X* valley, respectively, with scattering back into the Γ value occurring on a time scale of hundreds of fs to hundreds of ps, depending on phonon bottleneck processes.^37^ While they looked at InP bulk features, this correlates well with transitions from V1 to C1, C2, and C3/4, respectively, in our measurements. However, it has to be said that C1 and C2 seem to have larger cross sections in the experiment than expected from the calculated DOS. This may be explained on one hand by the fact that C1 and C2 states are bulk states that extend to the surface. Thus, there will be an accumulation of the contributions of many layers. Additionally, while some higher-energy states have larger DOS cross sections, the relative occupation level of near-CBM states is expected to be larger, as thermalizing electrons will accumulate there. This will increase emission signals relative to states such as C4, which are filled by fewer thermalization paths compared to C1/2. On the other hand, the methodological limitations of the present theory mentioned above may also contribute. This also matches bulk-InP resonance bands reported for In-rich InP.^25,27,62^ As after excitation there are electrons in these bulk bands, as well as a path for electrons to migrate to the surface, this is likely to cause the increased amplitude of C4 and C1/2 (states 5 and 2/3) relative to C3 and C5 (states 4 and 6), as shown in Figure 3. The latter would then appear to be filled mainly from thermalization of electrons at the p-InP surface rather than directly from bulk states directly. Considerations of whether states are assigned to surface or bulk features here are based on the results of DFT analysis, as shown in Figures 7 and 8, and thus are preliminary. In an upcoming paper, we explore the stability of peaks under surface modification and explore the assignment of surface *vs* bulk in more detail.

Combining experimental and theoretical results, we can now map the dynamics of an electron excited by a UV (4.49 eV) laser pulse; the results are shown in Figure 9. The electron decay paths are shown in red along the point of maximum emission from each state obtained from Figures 3 and 6.

The bulk state in Figure 8 refers to the bulk-to-surface transition (state 7). The electron occupation of the states is visualized in gray based on emission counts from the tr-2PPE transient, showing electrons thermalizing through the observed states after initial excitation.

## Conclusions

Our time-resolved two-photon photoemission spectroscopy study of P-rich InP(100) has revealed the presence of at least nine distinct states in the near-surface region. Among these states, we identified six unoccupied surface conduction band states (C1–C6), one bulk-to-surface transition state about 1.6 eV above the CBM, a surface defect state that pins the Fermi level, and a valence band state about 0.4 eV below the VBM.

The DFT calculations suggest C1, C2, and C4 to be particularly prone to electron migration from bulk to surface in addition to the bulk-to-surface transition state, which is correlated to their relative emission intensity relative to pure surface features. The energy of the bulk-to-surface transition displays scaling with photon energies between that of an occupied and unoccupied state and warrants further study. Furthermore, we successfully determined the decay constants of C1–C5, allowing us to track electron relaxation through the surface and bulk conduction bands.

This comprehensive understanding of the electron dynamics of the p-doped, P-rich, InP(100) surface for photon energies up to 4.5 eV provides a solid foundation for further exploration of the impact of surface modification by, for example, passivation layers on electron relaxation and transport processes. This opens new possibilities for enhancing the properties and applications of InP(100) by surface engineering.

## Supporting Information Available

Additional experimental details and methods, including schematics of the experimental setup and peak fitting procedure (PDF)

## Abbreviations

PEC: photoelectrochemistry
PV: photovoltaics
InP: indium phosphide
MOVPE: metalorganic vapor phase epitaxy
LEED: low-energy electron diffraction
Tr-2PPE: time-resolved two-photon photoemission spectroscopy
XPS: X-ray photoelectron spectroscopy
UPS: ultraviolet photoelectron spectroscopy
RAS: reflection anisotropy spectroscopy
TBP: tertbutylphospine
TMIn: trimethylindium
DEZn: diethylzinc
UHV: ultrahigh vacuum
DFT: density functional theory
DOS: density of states
