# Supplementary material for: Unraveling Electron Dynamics in p-type Indium Phosphide (100): A Time-Resolved Two-Photon Photoemission Study
Source: J Am Chem Soc. 2024 Mar 19;146(13):8949–60. doi: 10.1021/jacs.3c12487 (PMC10996002; doi:10.1021/jacs.3c12487)
Supplement: Supplementary file 1 — ja3c12487_si_001.pdf [file ja3c12487_si_001.pdf]

Unravelling Electron Dynamics in p-type Indium Phosphide (100): A Time-Resolved Two-Photon Photoemission Study

Jonathan Diederich[a,d], Jennifer Velasquez Rojas[a,d], Mohammad Amin Zare Pour[b], Isaac Azahel Ruiz Alvarado[c], Agnieszka Paszuk[b], Rachele Sciotto[c], Christian Höhn[a], Klaus Schwarzburg[a], David Ostheimer[b], Rainer Eichberger[a,b], Wolf Gero Schmidt[c], Thomas Hannappel[b], Roel van de Krol[a,d], Dennis Friedrich[a]*

[a] *Institute for Solar Fuels, Helmholtz-Zentrum Berlin für Materialien und Energie GmbH, 14109 Berlin, Germany*

[b] *Institut für Physik, Technische Universität Ilmenau, 98693 Ilmenau, Germany*

[c] *Lehrstuhl für Theoretische Materialphysik, Universität Paderborn, 33095 Paderborn, Germany*

[d] *Institut für Chemie, Technische Universität Berlin, 10623 Berlin, Germany*

ASSOCIATED CONTENT
SUPPLEMENTARY INFORMATION

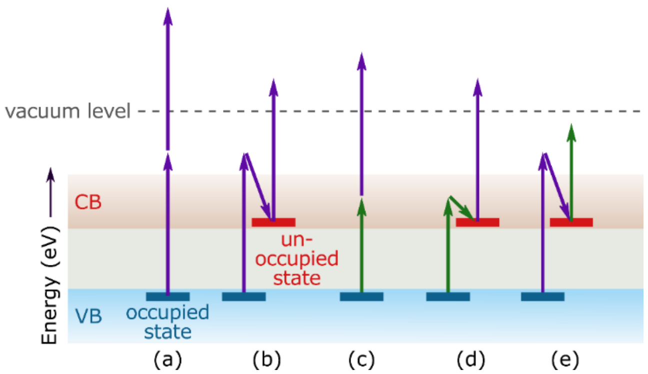

Fig. S1. Schematic of two-photon photoemission using two different wavelengths as pump or probe: 276 nm UV (violet) and 533 nm VIS (green). The length of the arrows is proportional to the photon energy. In a), two UV photons are incident simultaneously, in b) a UV photon photoexcites an electron, which decays down to the next-lowest state and is emitted from there by a second UV photon. c) and d) show a combination of two different photon energies. In c), a VIS photon pumps an electron which is emitted by a UV photon. In e) an electron is pumped by the UV photon, but then thermalizes too much energy before incidence of the VIS probe, and no emission of electrons is observed.

Supplementary Note 1:
Measurements were performed at room temperature (20.6 °C) in a UHV system at a base pressure of about 2 x 10^{-10} mbar. Laser pulses are generated in a Coherent Vitara Titanium Sapphire (Ti:Sa) oscillator pumped by a Verdi-G continuous wave laser. Subsequently, they traverse a grating-based compressor/expander unit before entering into a Coherent RegA 9050 amplifier pumped by a Verdi 12 laser. This results in pulses of 50 fs duration full-width half maximum (FWHM) at 800 nm peak wavelength and 150 kHz repetition rate with 9.5 µJ pulse energy. A schematic of the set-up is included below (Fig. S2 and Supplementary Note 2).
Two non-collinear parametric amplifiers (NOPA) are pumped through second harmonic generation in a β-barium borate (BBO) crystal. The first NOPA is usually set to produce a visible light (VIS) beam at 533 nm (2.33 eV) and 120 nJ pulse energy output, which is passed through a pair of quartz prisms to compensate the chirp of the pulses. This results in VIS pump pulses of 40 fs FWHM at the sample. The second NOPA is similarly tuned to 540 nm (2.30 eV) and 220 nJ pulse energy, with the beam also passing through a pair of prisms to compensate chirp. This beam is then frequency doubled to obtain a UV beam at 276 nm (4.49 eV) and 1.4 nJ pulse energy, which is split into a pump and a probe beam, both at 30 fs FWHM pulse width at the sample. The pulses are focused to spot sizes of the order of several tens of µm on the sample. Pulse lengths are determined by performing auto- (AC) and cross correlation (CC) measurements on a Cu (111) single crystal using the response of

a two-photon process from the occupied surface state mediated via a virtual intermediate state in the sp-band gap [1]. The copper sample is prepared beforehand by several sputtering (argon 5.0, 99.999% purity, Linde) and heating (527°C) cycles. The temporal overlap is achieved by scanning the time delay between the VIS or UV pump and UV probe beam across the region of increased emission caused by 2PPE, providing information on the FWHM of the pulses.

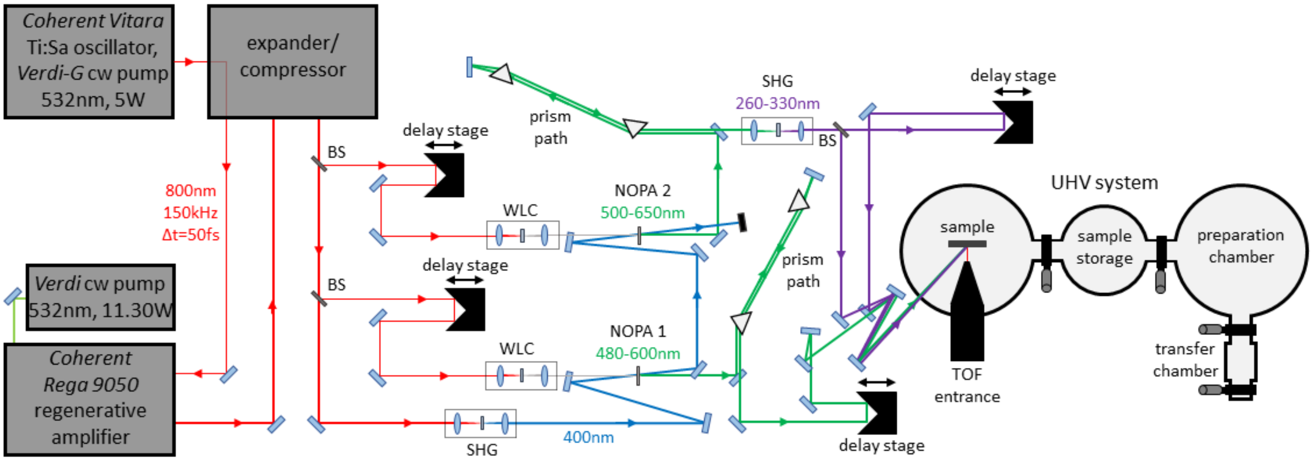

Fig. S2. Simplified schematic of the overall tr-2PPE set-up. The pulse-generating and -amplifying elements are on the left, NOPA 1 and 2 including 800 nm to 400 nm SHG in the middle. A prism path after each NOPA is shown, the output of NOPA 1 serves as the VIS pump, the output of NOPA 2 is frequency doubled in SHG and split to generate the UV pump and probe beams. All three beams are co-incident on the sample, which is placed in the UHV system, with the TOF detector placed 3-5 mm from the sample.

Supplementary Note 2:
Emitted electrons are detected using a time-of-flight (TOF) detector with a 7.3° acceptance angle, positioned normal to the sample surface at a distance of 3-5 mm. A larger distance between the sample and the detector entrance would restrict angular acceptance of emitted electrons and weaken the signal; a shorter distance would increase signals from electrons reflected of and scattered in the sample, thus lowering signal quality. The detector has an energy resolution of ca. 50 meV, with an uncertainty in photon energy of similar magnitude. During measurements, pump and probe beams are incident on the sample at an angle of 45°. To maintain reliable measurements, the pulse energies are constrained to specific values: 0.27 nJ for the pump and 0.13 nJ for the probe at 276 nm, or 3.3 nJ for the pump at 540 nm and 0.07 nJ for the probe at 276 nm. These limitations are imposed to prevent the build-up of a space charge region in front of the TOF detector and to avoid saturation and changes in surface band bending [2]. The density of photo-generated electrons is kept below 10^{17} cm^{-3} to avoid damaging the detector.

Supplementary Note 3:
Prior to transfer of the sample from the MOVPE reactor to the UHV transfer shuttle, the surfaces of the samples were characterized by RAS. The RA spectrum (Fig. S3) shows the characteristic 'optical fingerprint' of the P-rich InP(100) surface [6,7,8]. Presence of P at the surface was verified by XPS measurements of the P 2p core-level, displayed in Fig. S4 in the survey spectrum. The survey spectrum and the core-levels of O 1s and C 1s were measured at 90° photoelectron take-off angle (black lines). The core-level of P 2p in addition was measured at 30°, a more surface sensitive measurement, displayed in (Fig. S4, top right, orange line). Only a small amplitude of the O 1s and C 1s core-level peaks is visible (see high-resolution spectra in the top row), most probably absorbed to the surface during the transfer from the MOVPE reactor to UHV-chamber, no other contaminants are present on the surface. In the P 2p core-level (Fig. S4, right), two components can be fitted, each with spin-orbit splitting (the ratio of $2p_{3/2}$ and $2p_{1/2}$ was fixed to 2:1, and the FWHM of each peak was equal). The larger component is related to In-P bonds. The smaller surface component, which is shifted by 0.5 eV above binding energy (B.E.) of the In-P component, is related to P-P dimers on the surface [9]. The relative contribution of the P-P component increases when the photoelectron take-off angle (with respect to surface plane) is varied from 90° to 30°, **not shown here.** This confirms that the smaller component (red line) is a (near) surface component, while the In-P component stems from the bulk.

As shown by Vogt et al. experimentally, the p(2×2) and c(4×2) surface domains on the P-rich InP surface are of sizes on the order of nm [10]. XPS/UPS, LEED and tr-2PPE all use spot sizes several orders of magnitude larger, therefore no inaccuracies due to surface inhomogeneities are expected. Different spot positions and samples were used for all methods to verify reproducibility. In LEED, the observed (2×1) pattern is

the result of averaging over both p(2×2) as well as c(4×2) domains [10,11]. As small amounts of contamination, or incorrect synthesis of the P-dimer surface will lead to a simpler (1×1) LEED pattern instead, this was used as an initial verification of sample quality.

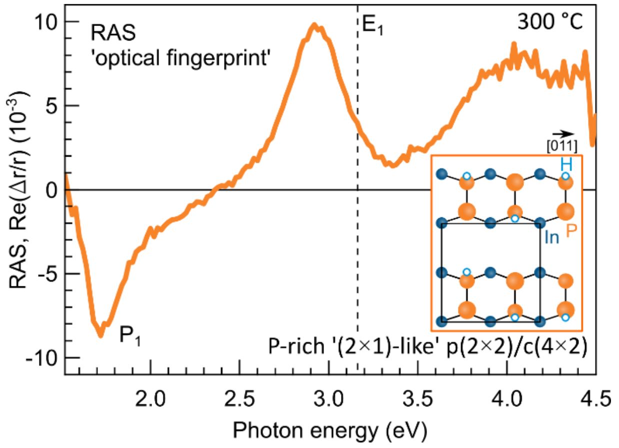

Fig. S3. The characteristic RA spectrum of the P-rich InP(100) surface at 300 °C, measured in the MOVPE reactor, prior to UHV transfer . This characteristic 'optical fingerprint' of the P-rich InP(100) surface shows a minimum at 1.9 eV (P_1). According to ab initio DFT calculations, this minimum is associated with optical transitions between occupied and unoccupied surface states caused by the H-termination of one H atom per P dimer [3,4,5]. The surface-modified bulk transitions cause the maximum at 3.2 eV, which is close to the E_1 interband transition.

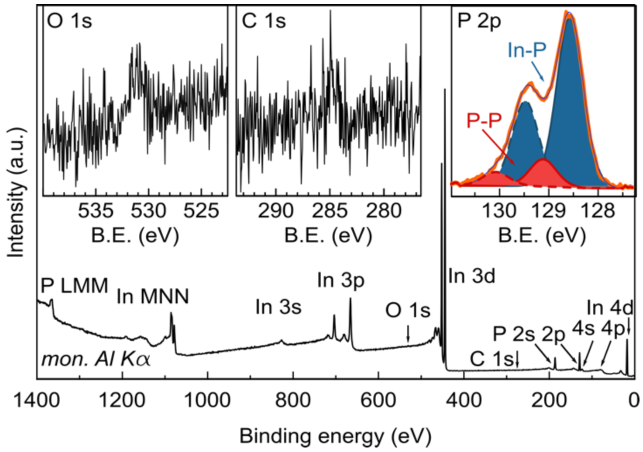

Fig. S4. The XPS survey spectrum of the corresponding sample (bottom), as well as enlarged binding energy regions of core-levels of O 1s, C 1s and P 2p (from left to right) are given, which were measured at a 90° (black lines) and at a 30° (shown only for P 2p, orange line) photoelectron take-off angle. In the core-level of the P 2p peak, the fit envelope is indicated by a blue line; it consists of components related to the In-P bonds (dark blue lines) and P-P bonds (red lines), which are solid for $2p_{3/2}$ and dashed for the $2p_{1/2}$ components.

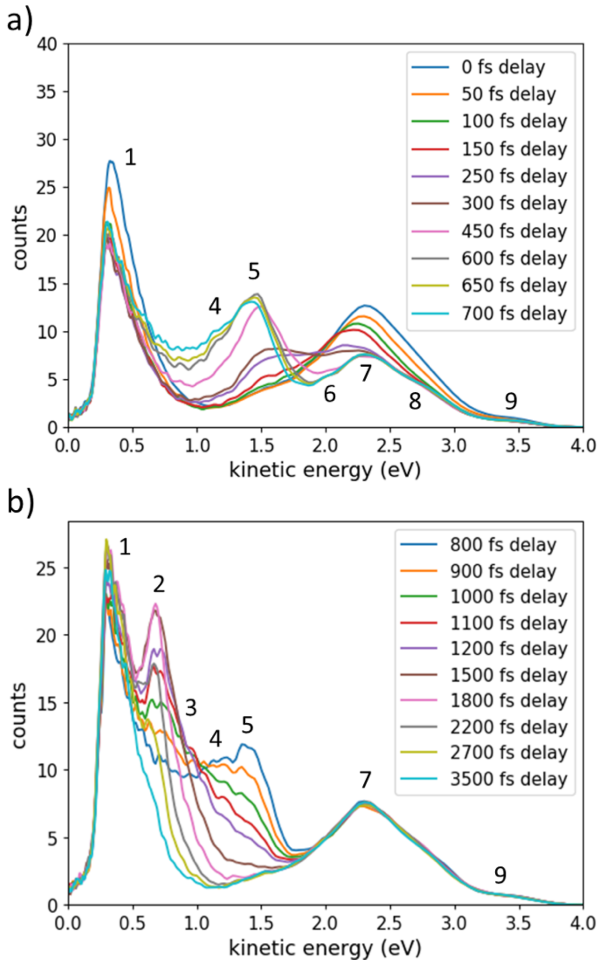

Fig. S5. More detailed delay slices for the tr-2PPE data in Fig. 3 are given, for the delay range up to 700 fs in (a), and for larger delay times in (b). Visible states are labelled for each delay range.

Supplementary Note 4:
Principal component analysis (PCA) was employed to separate overlapping states visually in Fig. S6. PCA in this case is performed using a Python script which takes the background-subtracted spectra from Fig. S6, and arranges them into a matrix, where each row represents a spectrum at a given delay time. The kinetic energy of a data point is the independent variable then, and the emission amplitude for that data point the dependent variable. The amplitudes for each kinetic energy are normalized, to ensure each variable contributes equally, and small effects are not overlooked.

The covariance matrix of the data is then computed, its elements describe the extent to which emission counts at two different kinetic energies vary together, or independently from each other. Eigenvalue decomposition of the covariance matrix is performed, where directions of maximum variance in the data (principal components) are identified; the eigenvalues then describe the magnitude of variance in the directions of the associated eigenvectors. Based on the eigenvalues, the contribution of each eigenvector to the overall variance is determined, and the eigenvectors are rank ordered.

By multiplying the matrix of selected eigenvectors with the original data, the data is transformed to a new space, defined by the principal components. Whilst the principal component eigenvectors themselves quite abstract, the projection onto the original data results in the outputted principal components still being given with kinetic energy on the x-axis. This allows for much more straightforward interpretation of data as compared to other methods such as singular value decomposition (SVD), where new x-axes are defined. A detailed outline of PCA can be found by Joliffe and Cadima at ref [12].

To summarize, PCA reduces the dimensionality of the dataset by reducing it to a set of orthogonal components that capture the most significant variance within the dataset, allowing for the analysis of complex spectral data by visual analysis. By identifying these principal components, PCA

can help uncover correlated changes in spectra, or reveal underlying physical processes, such as shared relaxation pathways or coupling between electronic states. It also allows for peaks which visually overlap in the raw data, but are filled and emptied via different mechanisms, to be separated visually. If two such adjacent and overlapping emission peaks in the raw data have a difference in temporal development of their emission counts, PCA will tend to separate them into different principal components, significantly increasing our ability to visually tell apart such overlapping peaks.

Peaks which appear as part of the same principal component in PCA are related; if e.g. two peaks both have positive amplitudes within the same principal component, they vary together across delay times. Possible reasons for this include shared excitation mechanisms, coupled states, or similar response to an external stimulus, such as the incidence of a pump pulse. If another peak within the same component has negative amplitude, then it is anti-correlated to the peaks of positive amplitude. In other words, when the peak at negative amplitude empties, the peaks at positive amplitude fill, or vice versa. This could be this state filling the other two or the other way around, alternatively it could indicate a competing pathway for electron relaxation.

In our case, states 3 (C2) and 5 (C4) are shown together in the second component of PCA in Fig. S8 (b), with a dip at the bulk-to-surface transition. This may indicate that both of these states fill through the bulk-to-surface transition cluster of states. In (a) C1 is visible as a peak, and C4 as a dip, indicating that thermalization through C4 provides a significant fraction of electrons to C1, whilst only a weak correlation to the bulk-to-surface transition is observed. This is particularly interesting, as it implies that phonon bottlenecking due to intervalley scattering to the *X*-point, correlated to C4 for bulk InP, may slow thermalization of carriers to C1.

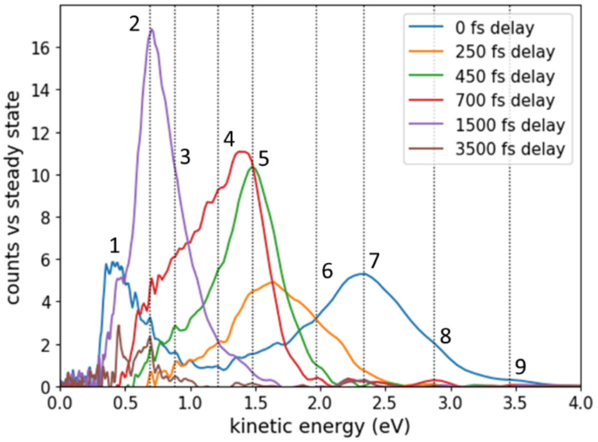

Fig. S6. The time-dependent component of tr-2PPE spectra from Fig. 3 is plotted by subtracting data at 5000 ± 1000 fs as a steady-state background. Vertical lines denote visible peaks corresponding to previously observed states at kinetic energies of 0.69, 0.88, 1.21, 1.48, 1.97, 2.33, 2.87 and 3.45 eV.

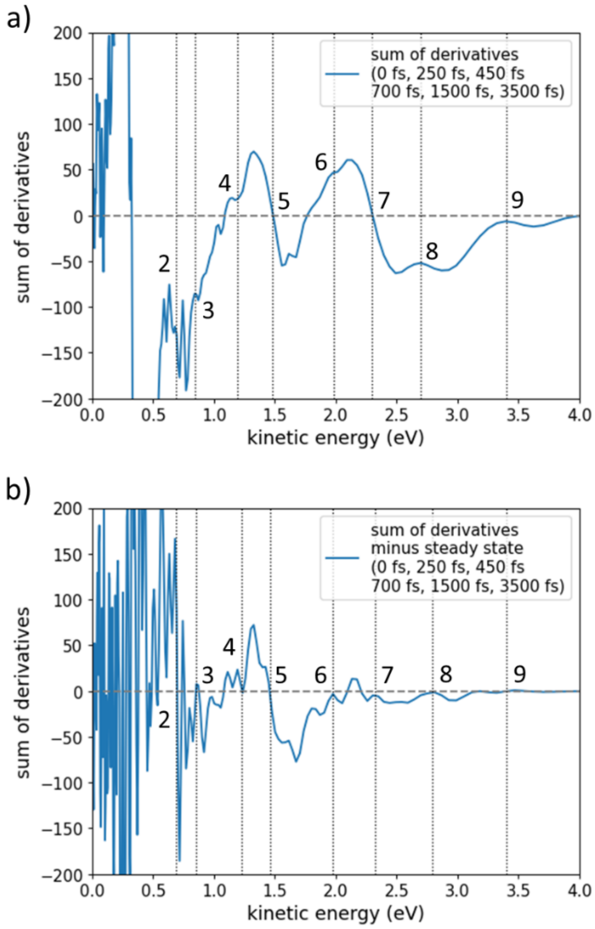

Fig. S7. The derivatives of counts from tr-2PPE spectra are summed up, this is given as a function of kinetic energy. Clear peaks in emission correlate to derivatives of 0 here, so points where the graph crosses the y-axis. Peaks within the shoulder of another peak show up as reduced sum derivatives at this point, so as a dip for positive derivatives or a peak at negative sum derivative values. Sum derivatives are given for the delay slices from Fig. 3 (b) here in (a), the same is given for the data with steady state emission subtracted (from Fig. S6) in (b). At low kinetic energies, the signal becomes noisy in (b), as the signal was largely time-independent there, and thus time-dependant signals are outweighed by noise, which has large sum gradients. Previously identified states 1-9 are shown as vertical dotted lines, with positions adjusted slightly to fit features here. States in (a) are given at 0.69, 0.84, 1.19, 1.48, 1.98, 2.30, 2.70 and 3.40 eV kinetic energy, with the background subtracted in (b) at 0.69, 0.85, 1.23, 1.46, 1.97, 2.32, 2.79 and 3.40 eV. This is in excellent agreement with values obtained from composite Voigt fitting, corroborating peak positions.

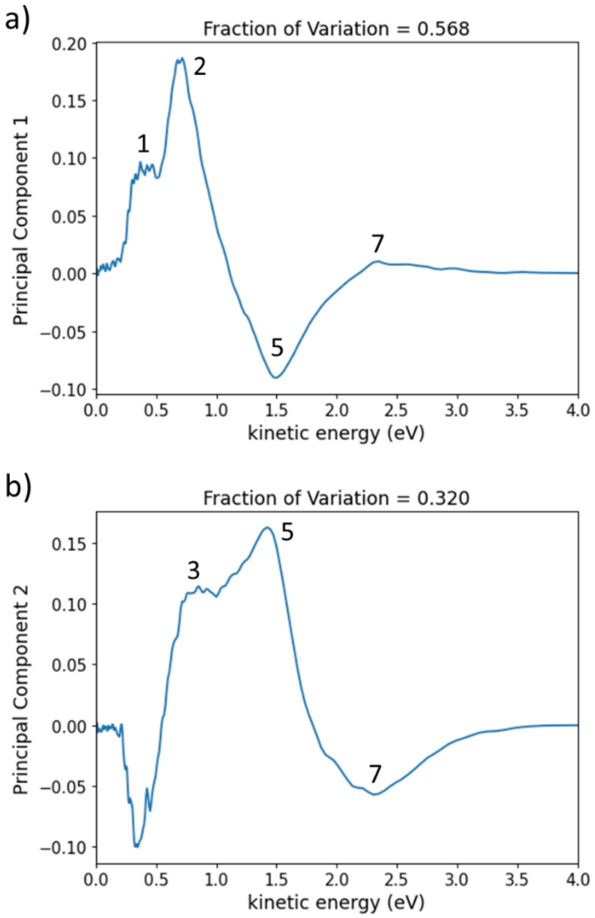

Fig. S8. Principal component analysis (PCA) is performed for the delay slices with background subtracted in Fig. S6; the x-axis remains as kinetic energy, allowing for straightforward interpretation of data. The principal two components from PCA are given in (a) and (b), accounting for 56.8% and 32.0% of variation respectively. Clear peaks are visible in (a) at 0.41 and 0.70 eV respectively, representing variation in emission from the surface state pinning the Fermi level, as well as state 2 (C1). In (b), peaks are clearly visible at 0.85 and 1.43 eV, corresponding to state 3 (C2) and state 5 (C4). This verifies especially the position of state 3 (C2).

Supplementary Note 5:

The composite Voigt fitting Python script (Fig. S9 and Fig. S10) uses Voigt peaks to represent both Gaussian and Lorentzian contributions to overall peak shapes in tr-2PPE.

The Python script has four fitted parameters for each peak: A, mu, sigma and gamma. A represents the amplitude, mu the position of the peak, sigma is used for the Gaussian, and gamma for the Lorentzian component of the peak. The Gaussian and Lorentzian components in the composite-Voigt script are determined via fitting. Sigma represents the standard deviation of the Gaussian component of the peak, a starting value of 0.1 eV is used. Gamma represents the half-FWHM of the Lorentzian component, and is also started at 0.1 eV for the fit. Starting values for A and mu were determined visually, and a range of values tried to ensure peaks are reliably fitted at identified state positions, and that fits are not simply a result of a specific choice of start values.

The Gaussian component represents several physical processes. One contribution is related to instrumental broadening related to the finite resolution of the TOF detector and other experimental components; this may vary with kinetic energy of the peak. Thermal broadening will also contribute, and as the temperature of excited carriers changes rapidly during thermalization, this component will change between peaks and delay times as well. Finally, defects, dislocations, impurities etc. will also add some Gaussian broadening. The Lorentzian component is caused in part by lifetime broadening of short-lived electronic states, which will vary across peaks and with the relative occupation of states. Many-body interactions are also a major factor, with energy and momentum exchange between excited carriers leading to a broadening of the observed energy distributions. This factor will highly vary with the excited carrier densities, which change with delay times and are different between states. As both the Gaussian and the Lorentzian components will vary between states, delay times, pump and probe intensities etc., we let the fit freely set sigma and gamma, rather than setting a fixed ratio between Gaussian and Lorentzian contributions.

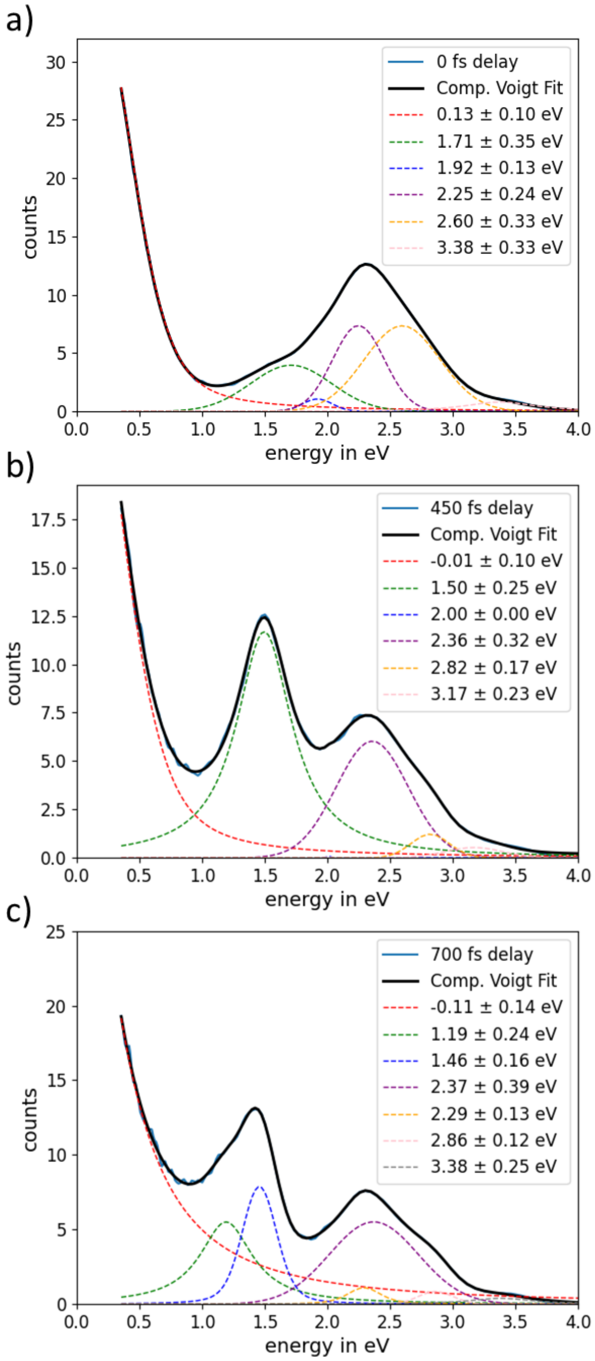

Fig. S9. Example composite Voigt fits of the P-rich InP(100) surface are given, with time delays taken at 0 fs (a), 450 fs (b) and 700 fs (c). Final state positions are determined by focusing on time delay slices at which the respective state is clearly visible, and averaging out kinetic energy values between slices. A proximity penalty is applied to avoid several peaks being fitted into a single feature.

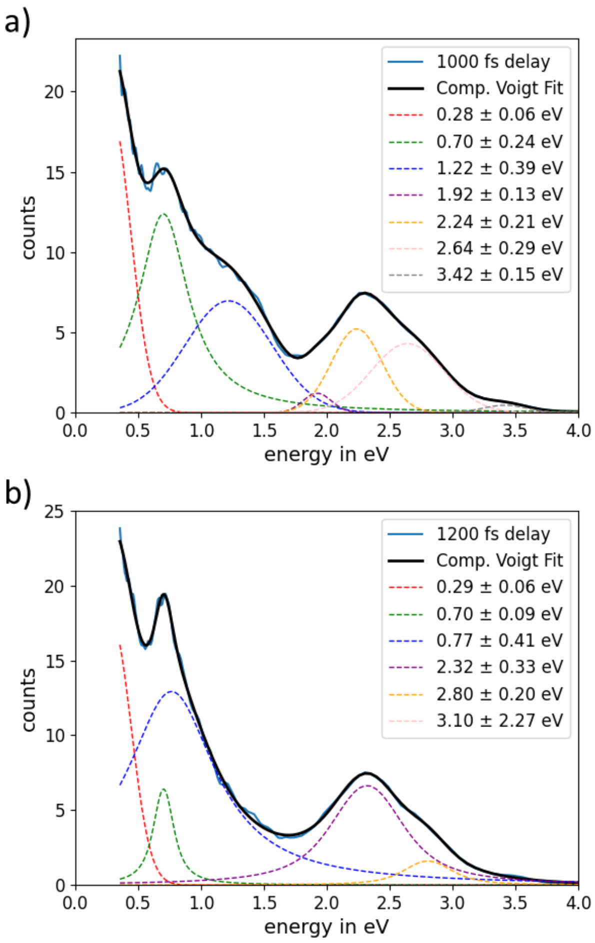

Fig. S10. Additional composite Voigt fits were performed at delay times other than those shown in Fig. 3 to verify state positions, for example at 1000 fs (a) and 1200 fs delay (b). Some states such as state 3 (C2) (corresponding to the blue line fit at 0.82 / 0.92 eV peak) or state 6 (C5) (yellow fit line with 1.83 / 2.06 eV peak) are less clear in position, corresponding to an increased uncertainty in state position as denoted in Table S1. However, fitting data at a range of delays and across samples indicate no clean fit to data is possible without including these features. Further corroboration for peaks is given above in Fig. S6, Fig. S7 and Fig. S8.

Supplementary Note 6:
Scaling of the kinetic energy of emitted electron with pump and probe energies is determined by whether a state is initially occupied or not. Direct two-photon photoemission from an occupied state such as in Fig.S11 (a) the change in kinetic energy equals two times the change in photon energy, for an unoccupied state as in Fig.S11 (b) the change in photon energy of the pump photon is thermalized away, thus kinetic energy scales with one time the photon energy.

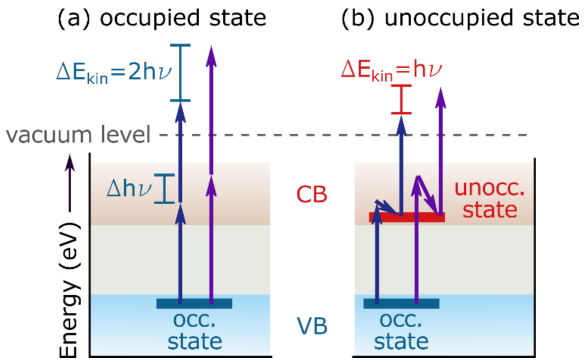

Fig. S11. Two-photon emission pathways are given for an occupied valence band (VB) state (a), and for an unoccupied conduction band (CB) state (b).

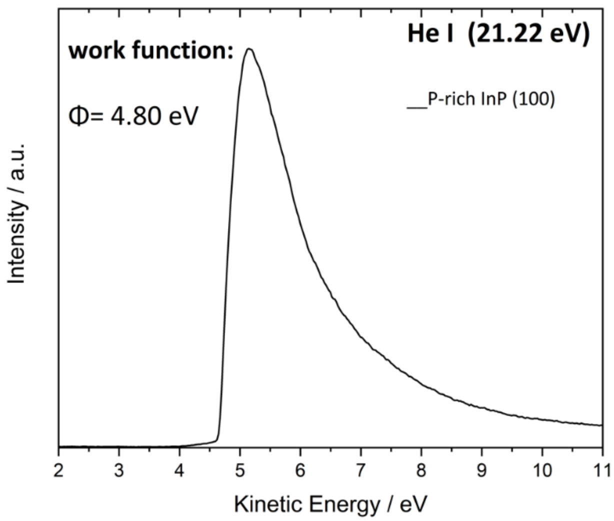

Fig. S12. Lower edge of emission of the p-InP surface in HE I UPS, the half-maximum position of the cut-off is taken to be the work function of the surface.

Peak position (eV) taken from UV-UV	Energetic position of associated state in surface (UV photons at 277 nm/4.48 eV in this measurement), $E_{state} = E_{kin}$ -4.48eV for C and bulk, $E_{state} = E_{kin}$ -2 x 4.48 eV for V1 and VBM	State number	Scaling with photon energy	Interpretation	Label
- 0.14 ± 0.05 combined UPS result	- 4.57 ± 0.05	1	-	surface state pinning the Fermi level	Fermi level
0.69 ± 0.05	- 3.79 ± 0.05	2	0.9 ± 0.1	conduction band state	C1
0.84 ± 0.10	- 3.64 ± 0.10	3	1.0 ± 0.2	conduction band state	C2
1.21 ± 0.10	- 3.27 ± 0.10	4	1.0 ± 0.2	conduction band state	C3
1.48 ± 0.05	- 3.00 ± 0.05	5	0.9 ± 0.1	conduction band state	C4
1.93 ± 0.10	- 2.55 ± 0.10	6	0.9 ± 0.1	conduction band state	C5
2.33 ± 0.05	- 2.15 ± 0.05	7	1.4 ± 0.1	bulk state	bulk state
2.77 ± 0.10	- 1.71 ± 0.10	8	1.1 ± 0.1	conduction band state	C6
3.40 ± 0.05	- 5.55 ± 0.05	9	2.1 ± 0.1	valence band state	V1
3.84 ± 0.05	- 5.11 ± 0.05	-	-	valence band edge	VBM

Table S1. Observed states are listed with their kinetic energy values in UV-UV, scaling of position with photon energy and the associated interpretation as conduction, valence or bulk states, with the resulting position relative to vacuum. The UV photon energy in this measurement was 4.48 eV, hence this is used instead of the nominal 4.49 eV for the sake of precision.

Supplementary Note 7:
An additional potential contribution to differences in lifetimes is related to diffusion of carriers due to surface band bending. For the p-InP surfaces used in this work, a previous paper by some of the present authors determined the density of charged surface states at 3.9×10^{12} cm^{-2}, causing the observed band bending. Given bulk doping of 2×10^{18} cm^{-3} and the requirement of charge neutrality, this leads to a charge accumulation layer thickness [13] of around 19.5 nm. For electrons diffusing towards this surface due to the charge gradient, this would define the diffusion length L_d. Given a diffusion constant D of 250 cm^{2}s^{-1} for InP [14], we determine the time constant τ_d associated with this diffusion [15]

$$\tau_d = \frac{L_d^2}{D} = \frac{(19.5\ nm)^2}{250\ cm^2 s^{-1}} \approx 15\ fs$$

This time constant is shorter than the UV and VIS pulse durations at around 30 and 40 fs respectively, indicating that the diffusion of carriers towards the surface purely due to band bending occurs near-instantaneously. For UV photons, the absorption depth in InP is about 12 nm, for VIS photons it is of the order of 100 nm [16]. Some of the electrons photoexcited at this depth will escape the material [17], and may contribute to slower diffusion in VIS. For $L_d \approx 100$ nm, the diffusion time τ_d becomes of the order of 400 fs. The increased decay constants for states in VIS- vs UV excitation may be related to this deeper absorption depth of the VIS-pulse, leading to gradual diffusion into surface states from the bulk, and thereby slowing observed thermalization. In either case, the diffusion time is less than the relaxation time of electrons, indicating that electrons at energies sufficient to occupy these discrete states, will also reach the surface before having thermalized away their excess energy above the CBM. This is a crucial basis for the design of hot-carrier cells, as it allows for electrons in the near-surface region to be collected at the surface via band-bending. Wu et al. [18] for InP quantum dot – methylviologen (MV^{2+}) complexes found charge transfer time scales on the order of 0.39 ps from the 1P state and above, this would be on the same order of magnitude as hot electron lifetimes in our data. Likely, carriers would however be extracted using a TiO_2 layer, in a follow-up paper we explore the electron dynamics with such protective and -charge separation layers on the p-InP surface described in the present work.

Supplementary Note 8:
Here, a word of caution is in order: The accuracy of DFT in describing excited states is limited. On the one hand, electronic self-energy effects tend to increase the excitation energies substantially. On the other hand, electron-hole attraction effects typically have a strong influence on the spectroscopic properties of an optically excited system. Both effects are strongly state specific and are not included in density-functional theory [19,20]. In the present work, they are for numerical reasons approximately accounted for by a rigid scissors shift of the unoccupied state energies. This approximation is hard to overcome for large systems, and will affect the predictive power of the calculations. The present calculations are certainly helpful for a qualitative interpretation of the experiment, but can methodologically not be expected to quantitatively reproduce the measured data. DFT calculations were performed after state numbers and -positions had been assigned, to avoid bias of the authors in interpreting the experimental data.

References:

(1) Hertel, T.; Knoesel, E.; Wolf, M.; Ertl, G. Ultrafast Electron Dynamics at Cu(111): Response of an Electron Gas to Optical Excitation. *Phys Rev Lett* **1996**, *76* (3), 535–538. https://doi.org/10.1103/PhysRevLett.76.535.

(2) Töben, L.; Gundlach, L.; Hannappel, T.; Ernstorfer, R.; Eichberger, R.; Willig, F. Dynamics of Electron Scattering between Bulk States and the C1 Surface State of InP(100). *Appl. Phys. A* **2004**, *78*, 239. https://doi.org/10.1007/s00339-003-2315-1.

(3) Schmidt, W. G.; Hahn, P. H.; Bechstedt, F.; Esser, N.; Vogt, P.; Wange, A.; Richter, W. InP(001)-(2×1) Surface: A Hydrogen Stabilized Structure. *Phys. Rev. Lett.* **2003**, *90* (12), 126101. https://doi.org/10.1103/physrevlett.90.126101.

(4) Hannappel, T.; Visbeck, S.; Töben, L.; Willig, F. Apparatus for Investigating Metalorganic Chemical Vapor Deposition-Grown Semiconductors with Ultrahigh-Vacuum Based Techniques. *Rev. Sci. Instrum.* **2004**, *75* (5), 1297–1304. https://doi.org/10.1063/1.1711148.

(5) Hahn, P. H.; Schmidt, W. G.; Bechstedt, F. Bulk Excitonic Effects in Surface Optical Spectra. *Phys. Rev. Lett.* **2001**, *88 1*, 016402.

(6) Hannappel, T.; Visbeck, S.; Knorr, K.; Mahrt, J.; Zorn, M.; Willig, F. Preparation of P-Rich InP Surfaces via MOCVD and Surface Characterization in UHV. *Appl. Phys. A* **1999**, *69* (4), 427–431. https://doi.org/10.1007/s003390051026.

(7) Letzig, T.; Schimper, H.-J.; Hannappel, T.; Willig, F. P–H Bonds in the Surface Unit Cell of P-Rich Ordered InP(001) Grown by Metalorganic Chemical Vapor Deposition. *Phys. Rev. B* **2005**, *71* (3). https://doi.org/10.1103/physrevb.71.033308.

(8) Döscher, H.; Möller, K.; Hannappel, T. GaP(100) and InP(100) Surface Structures during Preparation in a Nitrogen Ambient. *J. Cryst. Growth* **2011**, *318* (1), 372–378. https://doi.org/10.1016/j.jcrysgro.2010.10.132.

(9) Vogt, P.; Frisch, A. M.; Hannappel, Th.; Visbeck, S.; Willig, F.; Jung, Ch.; Follath, R.; Braun, W.; Richter, W.; Esser, N. Atomic Structure and Composition of the P-Rich InP(001) Surfaces. *Appl. Surf. Sci.* **2000**, *166* (1–4), 190–195. https://doi.org/10.1016/s0169-4332(00)00411-6.

(10) Vogt, P.; Hannappel, Th.; Visbeck, S.; Knorr, K.; Esser, N.; Richter, W. Atomic Surface Structure of the Phosphorous-Terminated InP(001) Grown by MOVPE. *Phys Rev B* **1999**, *60* (8), R5117–R5120. https://doi.org/10.1103/PhysRevB.60.R5117.

(11) Kleinschmidt, P.; Döscher, H.; Vogt, P.; Hannappel, T. Direct Observation of Dimer Flipping at the Hydrogen-Stabilized GaP(100) and InP(100) Surfaces. *Phys Rev B* **2011**, *83* (15), 155316. https://doi.org/10.1103/PhysRevB.83.155316.

(12) Jolliffe, I. T.; Cadima, J. Principal Component Analysis: A Review and Recent Developments. *Philos. Trans. R. Soc. Math. Phys. Eng. Sci.* **2016**, *374* (2065), 20150202. https://doi.org/10.1098/rsta.2015.0202.

(13) Lüth, H. Space-Charge Layers at Semiconductor Interfaces. In *Solid Surfaces, Interfaces and Thin Films*; Lüth, H., Ed.; Springer International Publishing: Cham, 2015; pp 337–391. https://doi.org/10.1007/978-3-319-10756-1_7.

(14) Adachi, S. Optical Dispersion Relations for GaP, GaAs, GaSb, InP, InAs, InSb, AlxGa1−xAs, and In1−xGaxAsyP1−y. *J. Appl. Phys.* **1989**, *66* (12), 6030–6040. https://doi.org/10.1063/1.343580.

(15) Sze, S. M.; Ng, K. K. *Physics of Semiconductor Devices*; 2006.

(16) Aspnes, D. E.; Studna, A. A. Dielectric Functions and Optical Parameters of Si, Ge, GaP, GaAs, GaSb, InP, InAs, and InSb from 1.5 to 6.0 eV. *Phys Rev B* **1983**, *27* (2), 985–1009. https://doi.org/10.1103/PhysRevB.27.985.

(17) Seah, M. P.; Dench, W. A. Quantitative Electron Spectroscopy of Surfaces: A Standard Data Base for Electron Inelastic Mean Free Paths in Solids. *Surf. Interface Anal.* **1979**, *1* (1), 2–11. https://doi.org/10.1002/sia.740010103.

(18) Wu, K.; Song, N.; Liu, Z.; Zhu, H.; Rodríguez-Córdoba, W.; Lian, T. Interfacial Charge Separation and Recombination in InP and Quasi-Type II InP/CdS Core/Shell Quantum Dot-Molecular Acceptor Complexes. *J. Phys. Chem. A* **2013**, *117* (32), 7561–7570. https://doi.org/10.1021/jp402425w.

(19) Bechstedt, F. *Many-Body Approach to Electronic Excitations: Concepts and Applications*; Springer Series in Solid-State Sciences; Springer Berlin Heidelberg, 2014.

(20) Hahn, P. H.; Schmidt, W. G.; Bechstedt, F. Bulk Excitonic Effects in Surface Optical Spectra. *Phys. Rev. Lett.* **2001**, *88 1*, 016402. https://doi.org/10.1103/PhysRevLett.88.016402.
